# Developing carbon assimilation methods in duckweed for insights into photosynthesis and growth mechanisms

**DOI:** 10.1093/plphys/kiag311

**Published:** 2026-05-26

**Authors:** Robert C Rintoul, Alison R Gill, Lorna McAusland, Erik H Murchie, Matthew Gilliham, Jenny C Mortimer

**Affiliations:** Australian Research Council Centre of Excellence in Plants for Space, Australia; School of Agriculture, Food and Wine and the Waite Research Institute, Adelaide University, Urrbrae, South Australia, Australia; Division of Plant and Crop Sciences, School of Biosciences, University of Nottingham, Sutton Bonington, Leicestershire, United Kingdom; Australian Research Council Centre of Excellence in Plants for Space, Australia; School of Agriculture, Food and Wine and the Waite Research Institute, Adelaide University, Urrbrae, South Australia, Australia; Division of Plant and Crop Sciences, School of Biosciences, University of Nottingham, Sutton Bonington, Leicestershire, United Kingdom; Australian Research Council Centre of Excellence in Plants for Space, Australia; Division of Plant and Crop Sciences, School of Biosciences, University of Nottingham, Sutton Bonington, Leicestershire, United Kingdom; Australian Research Council Centre of Excellence in Plants for Space, Australia; School of Agriculture, Food and Wine and the Waite Research Institute, Adelaide University, Urrbrae, South Australia, Australia; Australian Research Council Centre of Excellence in Plants for Space, Australia; School of Agriculture, Food and Wine and the Waite Research Institute, Adelaide University, Urrbrae, South Australia, Australia

## Abstract

Infrared gas analysis (IRGA) is the primary technique for quantifying CO_2_ uptake in plants, but technical obstacles have limited its application in aquatic species, inhibiting exploration of their photosynthetic mechanisms. Using an IRGA with a customized aquatic chamber, we developed a methodology for measuring CO_2_ responses in duckweed, a group of fast-growing, floating angiosperms. We compared three morphologically contrasting species that have commercial or environmental significance: *Wolffia australiana, Spirodela polyrhiza*, and *Lemna minuta*. Light response curves showed species variation in light saturation intensity and light-saturated rates of photosynthesis, with *W. australiana* generally showing the highest rates of exchange per unit area under CO_2_-limited and CO_2_-saturated conditions. We estimated biochemical parameters (*V*_cmax_, *J*_max_) from CO_2_ response curves, though direct measurement of conductance and intercellular CO_2_ (*C*_i_) was not possible. Using estimated *C*_i_, predicted *V*_cmax_ and *J*_max_ values fell within ranges consistent with other angiosperms, and again *W. australiana* had the highest values. Temperature response curves suggested an optimum of 35 °C. Combining gas exchange and chlorophyll fluorescence further revealed a higher photosynthetic performance in *W. australiana* grown at lower plant densities. The suite of methodologies presented here will enable future mechanistic studies of duckweed carbon assimilation, growth dynamics and environmental responses, advancing understanding to levels comparable with that of many other terrestrial plant species.

## Teaser

Gas exchange methods were developed for duckweeds, fast-growing, floating aquatic plants, to enable mechanistic studies of carbon assimilation, growth and environmental responses across genera.

## Introduction

Duckweed species are C_3_ aquatic floating plants within the family Araceae. Under ideal conditions they can double their biomass in 2 to 4 days (Yu et al. 2014; Ziegler et al. 2015). This high growth rate is due to their simple body plans that consist of a reduced structure called a frond, which is most similar to a leaf (distinct upper and lower layers, stomata and chloroplasts), stem (meristematic region), and root (nutrient absorption) in one tiny package (Ware et al. 2023; Ziegler et al. 2023). There are 5 genera of duckweed grouped into 2 subfamilies (number of species belonging to each in parentheses), the rooted Lemnoideae, which includes *Spirodela* (2), *Landoltia* (1), and *Lemna* (12), and the rootless Wolffioideae, which includes *Wolffiella* (10) and *Wolffia* (11) (Les et al. 2002; Bog et al. 2020). Among the 32 species are some of the world's smallest flowering plants. However, duckweeds do not readily flower and instead most commonly reproduce asexually, generating populations of genetically uniform clones at a growth rate beyond that of other higher plants, making them the fastest-growing angiosperms on Earth (Sowinski et al. 2019).

Alongside their compact size and substantial potential yield, they have a remarkable tolerance to abiotic stresses, including high temperatures, extreme light and pH, and contaminated water (Stewart et al. 2021; Ziegler et al. 2023). These qualities make duckweed attractive for economic applications in phytoremediation, biomanufacturing, and biofuels and as a protein-rich food source (Ofoedu et al. 2025). Duckweed has also been proposed to be an interesting candidate to support bioregenerative systems during deep space missions (Romano and Aronne 2021; Morgan et al. 2024). Despite duckweed's exceptional ability to convert light into biomass and survive over a wide range of environmental conditions, a historic lack of tools has limited full exploration of duckweed carbon assimilation and photosynthetic mechanisms.

Aquatic plants require a distinct approach to gas exchange analysis in comparison to aerial leaf tissue due to gas diffusion through the liquid media phase. Duckweeds with floating fronds have both an aquatic and aerial component, compounding the problem. Noncontact approaches such as chlorophyll fluorescence and hyperspectral reflectance are readily deployable (Smith et al. 2024), but to understand stress and environmental responses and elucidate tolerance mechanisms in the context of photosynthetic carbon assimilation and growth rates, CO_2_ and water flux measured by infrared gas analysis (IRGA) is required. This will enable mechanistic studies of photosynthetic limitations that have advanced our understanding of terrestrial species, including crops. To date, this type of approach has not been possible for duckweed.

Fundamental questions also remain about the limitations to duckweed photosynthesis and how the observed high growth rates are supported. An important goal is to decouple components of control by Rubisco, electron transport, CO_2_ diffusion and photoprotection. For example, a unique feature of duckweed stomata is that they are commonly observed to be fully open and nonresponsive (Hoang et al. 2019; Ernst et al. 2025), and so CO_2_ limitation by stomatal conductance has been assumed to be low, but this needs to be tested under diverse temperature and light conditions, which requires the development of a tractable IRGA system with chlorophyll fluorescence. Duckweed stomata are located on the upper surface, with spherical stomata in *Spirodela* and *Lemna* species and elliptical ones in *Landoltia*, *Wolffiella*, and *Wolffia* species (Hoang et al. 2019). Stomata are usually observed to be open (Hoang et al. 2019), and stomatal density differs between species, with *S. polyrhiza*, *L. punctata*, and *L. minor* having 290.1, 210.7, and 97.5 stomata/mm^2^, respectively (Fang et al. 2025). Stomatal aperture is approximately 6.5 μm in all 3 species. The ability to perform *A/C*_i_ measurements would represent a step change in understanding duckweed photosynthetic control, and further research into stomatal characteristics is warranted.

Recently, photosynthetic parameters have been measured on some aquatic species, including algae (Hupp et al. 2021), seaweed, and coral (Davey and Lawson 2024), using a LI-6800 portable photosynthesis system fitted with an aquatic chamber (6800-18; LI-COR 2022b). There have yet to be any peer-reviewed reports of its use for aquatic vascular plants. Loew-Mendelson et al. (2025) measured CO_2_ assimilation in *Elodea canadensis* and *Egeria densa*, 2 fully submerged vascular plants, but floating plants like duckweed have not yet been investigated with IRGA. Previous limitations in measuring aquatic samples included the need for dry air to enter the sample chamber to prevent optical path contamination and the large water vapor differential, which exceeded linearity and reduced instrument accuracy (Davey and Lawson 2024). Coupled with technological limitations, the use of IRGAs for photosynthesis in aquatic or semiaquatic floating plants has therefore not been possible until now.

Here, we developed best-practice methods to obtain gas exchange measurements in duckweed fronds across diverse duckweed species. We describe the principles and assumptions needed for the application to the unique scenario of aquatic floating plants; standardized gas exchange methods to enable future duckweed studies on photosynthetic parameters, including responses to light, CO_2_ concentration, and temperature; and the potential of combining this system with chlorophyll analysis methods. Challenges remain, however, such as direct measurement of stomatal conductance.

## Results

### Development of methods to measure gas exchange in duckweed

**Figure 1:**
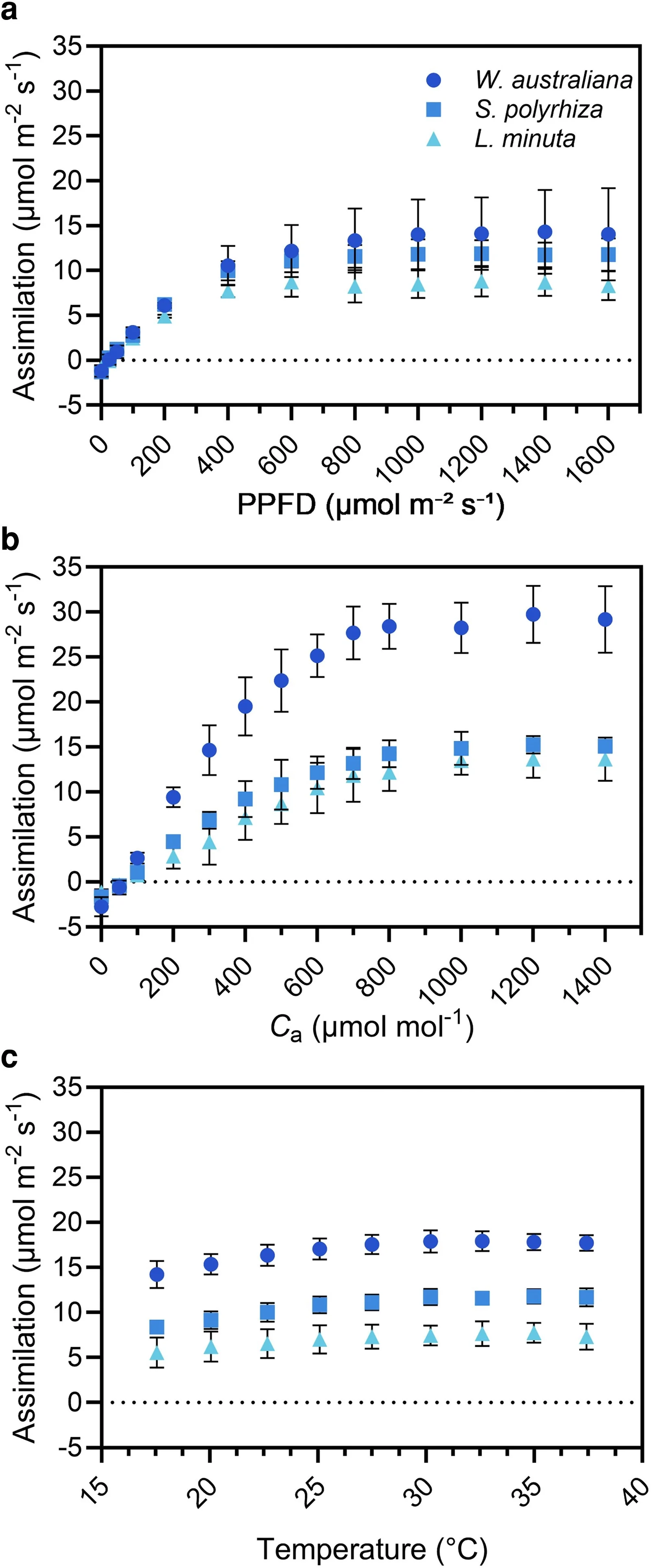
Assimilation as a function of increasing PPFD (a), CO_2_ concentration (b), and temperature (c) in 3 duckweed species: *W. australiana*, *S. polyrhiza*, and *L. minuta*. Results were normalized to area. For the light response curve (a), data are means ± SD (n = 5). Measurements were made at a CO_2_ reference concentration of 400 µmol mol^−1^. For the CO_2_ response curve (b), *C*_a_ = CO_2_ concentration data are mean ± SD (n = 3–4). Measurements were made at a saturating PPFD of 1,000 µmol m^−2^ s^−1^. For the temperature response curve (c), data are means ± SD (n = 3). Measurements were made at a saturating PPFD of 1,000 µmol m^−2^ s^−1^ and a CO_2_ reference concentration of 400 µmol mol^−1^.

We developed gas exchange methods to measure the CO_2_ assimilation of 3 duckweed species, *W. australiana*, *S. polyrhiza*, and *L. minuta*, in response to changes in PPFD (Fig. 1a), CO_2_ concentration (Fig. 1b), and temperature (Fig. 1c). All curves followed the characteristic nonrectangular hyperbola expected of higher plant CO_2_ assimilation (Busch et al. 2024).

For light response curves, the R modeling package *photosynthesis* (Stinziano et al. 2021, 2023; Marshall and Biscoe 1980) was used to calculate the PPFD at which 90% of the theoretical maximum assimilation occurs (LSP_90%_) and at which CO_2_ assimilation would theoretically no longer be light-limited (Figure S1). *W. australiana* saturates at 925 µmol m^−2^ s^−1^, a much higher PPFD than *S. polyrhiza* and *L. minuta* (LSP_90%_ of 558 and 358 µmol m^−2^ s^−1^, respectively) (Fig. 1a). CO_2_ assimilation at saturating PPFD (*A*_sat_, measured at ambient CO_2_) was significantly greater for *W. australiana* when compared with *L. minuta* (16.43 and 9.28 µmol m^−2^ s^−1^, respectively), with both species not statistically different from *S. polyrhiza* (13.15 µmol m^−2^ s^−1^) (Table 1). Light compensation points were not significantly different between the species, ranging from 23 to 26 µmol m^−2^ s^−1^. Dark respiration ranged from 0.7 to 1.12 µmol m^−2^ s^−1^, with no significant differences between species.

**Table 1:** Summary of variables from light, CO_2_, and temperature response curves for *W. australiana*, *S. polyrhiza*, and *L. minuta* fit using the R *photosynthesis* package (Stinziano et al. 2023).

Curve	*Wolffia australiana*	*Spirodela polyrhiza*	*Lemna minuta*
Light response			
*A*_sat_, µmol m^−2^ s^−1^	16.43 ± 5.8^a^	13.15 ± 2.1^ab^	9.28 ± 1.7^b^
LCP, µmol m^−2^ s^−1^	26 ± 12^a^	23 ± 6^a^	23 ± 9^a^
LSP_90%_, µmol m^−2^ s^−1^	925 ± 389^a^	558 ± 181^ab^	358 ± 97^b^
*R*_d_, µmol m^−2^ s^−1^	1.12 ± 0.57^a^	0.96 ± 0.44^a^	0.7 ± 0.27^a^
CO_2_ response (*A/C*_i_)			
100% *C*_a_			
*V*_cmax,_ µmol m^−2^ s^−1^	59.79 ± 10.0^a^	29.62 ± 4.79^b^	22.5 ± 8.09^b^
*J*_max_, µmol m^−2^ s^−1^	157.4 ± 24.97a	73.93 ± 5.04^b^	60.13 ± 14.79^b^
CO_2max_, µmol mol^−1^	735 ± 176^a^	702 ± 229^a^	578 ± 96^a^
66% *C*_a_			
*V*_cmax_, µmol m^−2^ s^−1^	77.67 ± 13.08^a^	38.54 ± 7.23^b^	29.96 ± 10.0^b^
*J*_max_, µmol m^−2^ s^−1^	160.7 ± 24.59^a^	74.39 ± 5.15^b^	63.03 ± 13.35^b^
CO_2max_, µmol mol^−1^	364 ± 132^a^	409 ± 101^a^	472 ± 190^a^
33% *C*_a_			
*V*_cmax,_ µmol m^−2^ s^−1^	136.9 ± 32.52^a^	66.32 ± 14.48^b^	51.46 ± 12.87^b^
*J*_max_, µmol m^−2^ s^−1^	178.4 ± 30.7^a^	79.66 ± 8.84^b^	69.2 ± 9.19^b^
CO_2max_, µmol mol^−1^	160 ± 76^a^	210 ± 78^a^	269 ± 65^a^
Temperature response			
*A*_opt_, µmol m^−2^ s^−1^	17.97 ± 1.11^a^	11.81 ± 0.79^b^	7.61 ± 1.23^c^
T_opt_, °C	33.51 ± 2.0^a^	35.4 ± 2.7^a^	35.48 ± 2.8^a^

All data are means ± SD (light curves, n = 5; CO_2_ curves, *W. australiana* and *S. polyrhiza*, n = 3; CO_2_ curves, *L. minuta*, n = 4; temperature curves, n = 3). Different letters indicate significant differences among species for each parameter (*P* < 0.05; one-way ANOVA, Tukey multiple comparisons test). Species sharing at least one letter are not significantly different.

Abbreviations: *A*_opt_, assimilation at optimum temperature; *A*_sat_, assimilation at saturating PPFD; CO_2max_, CO_2_ concentration at saturation; *J*_max_, maximum rate of electron transport; LCP, light compensation point; LSP_90%_, 90% of the light saturation point; *R*_d_, dark respiration; T_opt_, optimum temperature; *V*_cmax_, maximum rate of Rubisco carboxylation.

CO_2_ response curves (Fig. 1b) were conducted at a PPFD of 1,000 µmol m^−2^ s^−1^, as this was saturating for all species without being excessive (Table 1). The mole fraction of CO_2_ at which carbon assimilation was determined to be no longer limited by CO_2_ (CO_2_ saturation point) was not significantly different between the 3 duckweed species, determined to be 735, 702, and 578 µmol mol^−1^ CO_2_, for *W. australiana*, *S. polyrhiza*, and *L. minuta*, respectively (Table 1).

With the current system, it is not possible to measure *C*_i_ in duckweed, so the maximum rate of Rubisco carboxylation (*V*_cmax_) and maximum rate of electron transport (*J*_max_) cannot be calculated directly. However, given that *C*_i_ typically represents a fraction of the atmospheric carbon dioxide (*C*_a_), it was possible to create *A*/*C*_a_ curves with estimated values for *C*_i_ to determine the potential magnitude of variation in *V*_cmax_ and *J*_max_. Using the *photosynthesis* package in R (Stinziano et al. 2023), *A/C*_a_ curves were fitted, based on Farquhar et al. (1980), at 100%, 66%, and 33% of *C*_a_. This yielded mean *V*_cmax_ ranges of 59.79 to 136.9 µmol m^−2^ s^−1^, 29.62 to 66.32 µmol m^−2^ s^−1^, and 22.50 to 51.46 µmol m^−2^ s^−1^ for *W. australiana*, *S. polyrhiza*, and *L. minuta*, respectively (Table 1). The estimated *V*_cmax_ for *W. australiana* was significantly greater than that for *S. polyrhiza* and *L. minuta* at all 3 assumed values of *C*_i_. Estimated *J*_max_ was also consistently significantly higher in *W. australiana* (157.4–178.4 µmol m^−2^ s^−1^) than both *S. polyrhiza* (73.93–79.66 µmol m^−2^ s^−1^) and *L. minuta* (60.13–69.20 µmol m^−2^ s^−1^) (Table 1).

As is standard practice, measurements were taken at ambient CO_2_ twice (between 0 and 500 µmol mol^−1^), once at the beginning of the curve and a second midway through. Given that duckweed stomata have been reported to be “constantly” open (Hoang et al. 2019; Ernst et al. 2025), it was assumed that the CO_2_ diffusion limitation to the sites of carboxylation remained constant. However, there was considerable variation between the assimilation values at the ambient setpoint (2–43%) within replicates. This disparity could be related to differences in stomatal aperture or biochemical limitations and did affect estimation of *V*_cmax_. We measured stomatal density in all 3 species, and there were no significant differences between species (mean: 170, 156, and 207 stomata mm^−2^ for *W. australiana*, *S. polyrhiza*, and *L. minuta*, respectively) (Table S1).

The temperature response of duckweeds was investigated using a recirculating water bath to cool and heat the aquatic chamber block (but not the sample well) alongside control of air temperature via a heat exchanger in the mixing fan volume. For measurements, a saturating PPFD (1,000 µmol m^−2^ s^−1^) was used and the CO_2_ concentration was controlled at 400 µmol mol^−1^. The temperature at which assimilation peaked (T_opt_) and the respective assimilation rate at T_opt_ (*A*_opt_) were determined across a range of temperatures (Hobbs et al. 2013) (Figure S2). The curve predicted maximum assimilation for *W. australiana* at 33.5 °C, which was lower, albeit not significantly, than the ∼35.5 °C predicted for *S. polyrhiza* and *L. minuta* (Fig. 1C and Table 1). *A*_opt_ was significantly greater in *W. australiana* (17.97 µmol m^−2^ s^−1^) than in *S. polyrhiza* (11.81 µmol m^−2^ s^−1^) and *L. minuta* (7.61 µmol m^−2^ s^−1^) (Table 1). Comparison between the temperature response curve measurements taken at 25 °C and the corresponding light curve data points reported no significant differences (absolute difference in CO_2_ assimilation, *P* value from unpaired *t* test with Welch's correction): *W. australiana* (3.04 µmol m^−2^ s^−1^; *P* = 0.1645), *S. polyrhiza* (0.99 µmol m^−2^ s^−1^; *P* = 0.3235), and *L. minuta* (1.45 µmol m^−2^ s^−1^; *P* = 0.2730).

### Comparing methods of normalization in duckweed assimilation data

**Figure 2:**
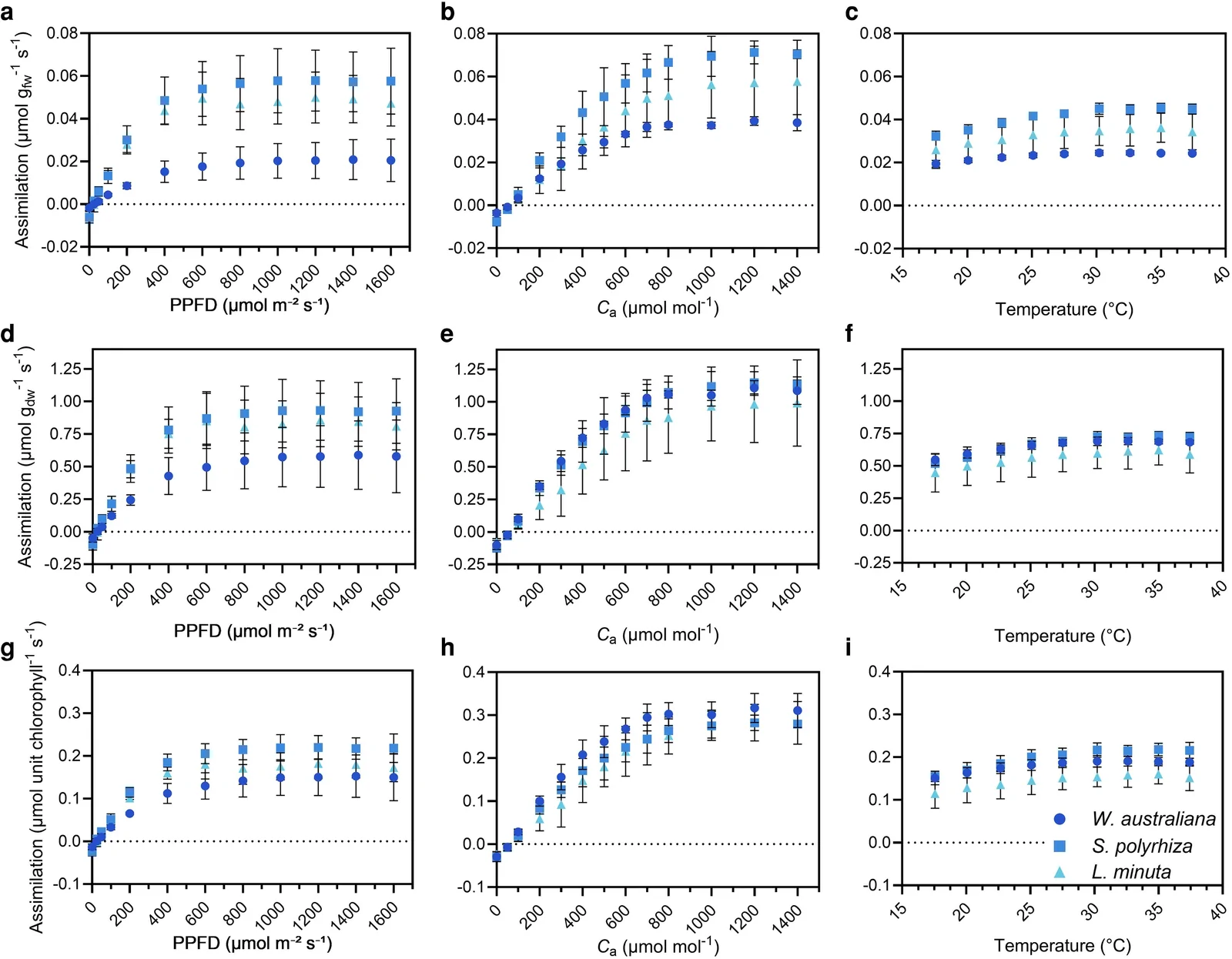
Assimilation normalized to fresh weight (a–c), dry weight (d–f), or total chlorophyll content (g–i) for 3 duckweed species: *W. australiana*, *S. polyrhiza*, and *L. minuta*. All data points are means ± SD. For light response curves (a, d, and g), measurements were made at a CO_2_ reference concentration of 400 µmol mol^−1^ (n = 5). For CO_2_ response curves (b, e, and h), measurements were made at a saturating PPFD of 1,000 µmol m^−2^ s^−1^ (n = 3–4). For temperature response curves (c, f, and i), measurements were made at a saturating PPFD of 1,000 µmol m^−2^ s^−1^ and at a CO_2_ reference concentration of 400 µmol mol^−1^ (n = 3). Abbreviation: *C*_a_, CO_2_ concentration.

Data were normalized to surface area (Fig. 1, Table 1), fresh and dry weight, and total chlorophyll content per square meter. For maximum assimilation rates, when normalized by area, *W. australiana* had the highest assimilation, but when normalized by weight, *S. polyrhiza* had the highest assimilation (Fig. 2a–2c). This is likely due to the surface area-to-weight ratio of each duckweed, where *S. polyrhiza* and *L. minuta* both have wider and thinner fronds compared with the small but thicker frond/pseudo root structure of *W. australiana*.

The variability in these measurements depends on how the values are expressed. *W. australiana* measurements show less variability when normalized to weight compared with area, perhaps due to weight yielding a more consistent measure of biomass compared with surface area due to a greater proportion of below-surface fronds. The inverse was true for rooted species, which showed higher variability when normalized to weight, perhaps due to lengths of roots (and thus weight) varying irregularly with frond area. Additionally, the calculation of area and thus normalization of Lemnoideae species cannot account for overlap between fronds, which are likely still assimilating CO_2_, but at a lower rate, thus potentially distorting the rates for *S. polyrhiza* and *L. minuta* on an area basis.

As total chlorophyll content was greatest in *W. australiana* (93.87 mg m^−2^), normalization of chlorophyll content reduced its CO_2_ assimilation in comparison to *S. polyrhiza* and *L. minuta* (53.99 mg m^−2^ and 48.29 mg m^−2^, respectively) (Fig. 2g–2i). Per unit chlorophyll, *S. polyrhiza* showed the greatest CO_2_ assimilation, particularly as a function of increasing CO_2_ concentration and temperature. When normalized to dry weight or chlorophyll content, differences in assimilation between duckweed species were reduced, particularly for the temperature response curves.

### Assimilation rates are stable throughout the day

**Figure 3:**
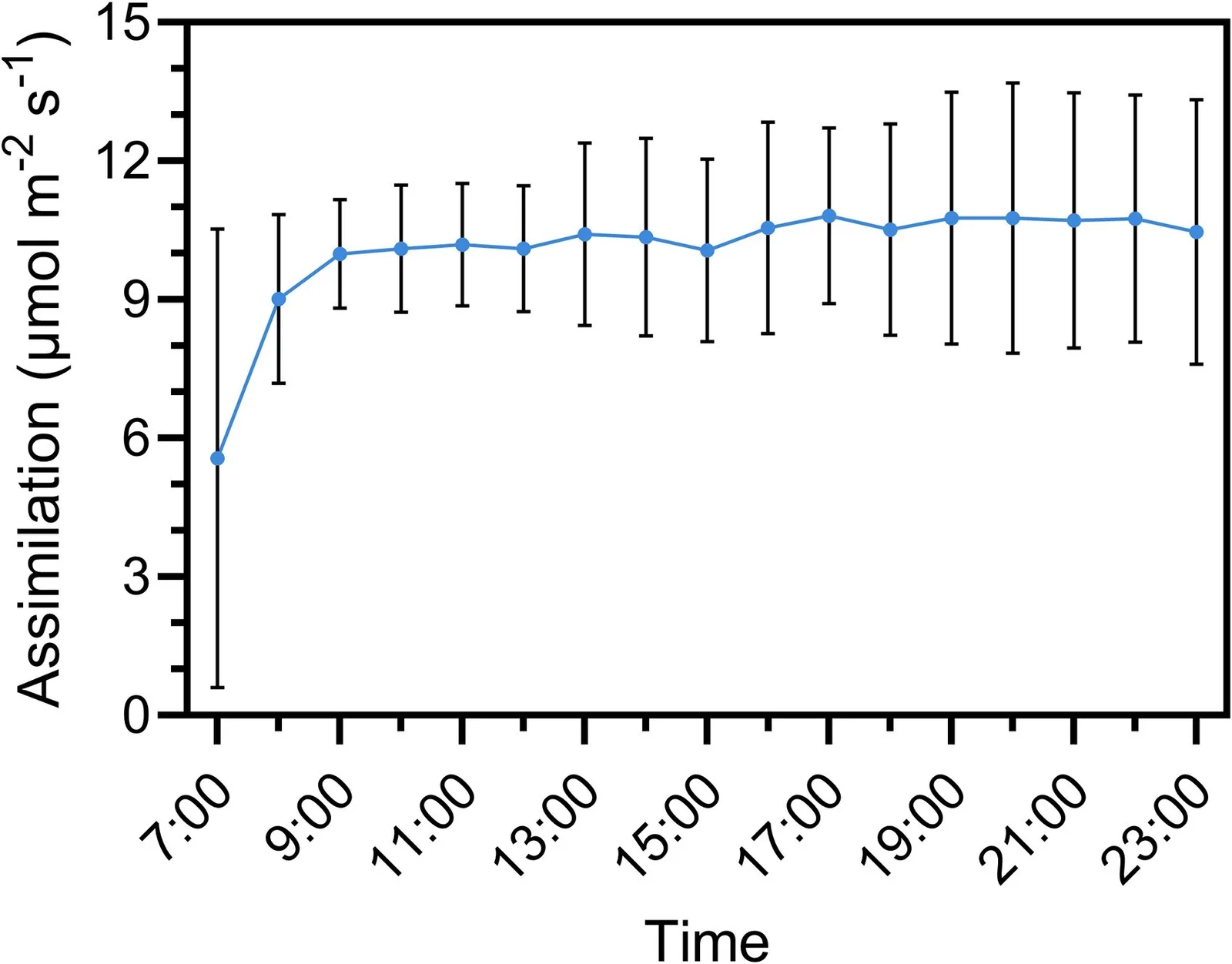
Diurnal curve of assimilation (normalized to area) for *S. polyrhiza*, taken from just before 7:00 AM at lights on until 11:00 PM at lights off. Measurements were made at a PPFD of 1,000 µmol m^−2^ s^−1^ (Q_sample_), *C*_a_ of 400 µmol mol^−1^, and logs were taken every 5 min but presented every hour. Data are mean ± SD (n = 3). Abbreviation: *C*_a_, CO_2_ concentration.

Next, fundamental features of duckweed growth relating to directly measured CO_2_ assimilation were explored. Duckweed CO_2_ assimilation was measured every 5 min over a diurnal period (16 h from lights on) in *S. polyrhiza* (Fig. 3). *S. polyrhiza* was chosen as it was easiest to move to and from the chamber, as well as its minimal variability between replicates in previous measurements. CO_2_ assimilation increased rapidly to a mean of 9.02 µmol m^−2^ s^−1^ in the first hour when lights were turned on, before reaching a steady state and remaining stable, between 9.99 and 10.8 µmol m^−2^ s^−1^, throughout the day. The only time point that was significantly different from the others was the initial measurement taken at 7:00 AM.

### Growth rates in duckweed are associated with measured assimilation, chlorophyll fluorescence, and frond density

**Figure 4:**
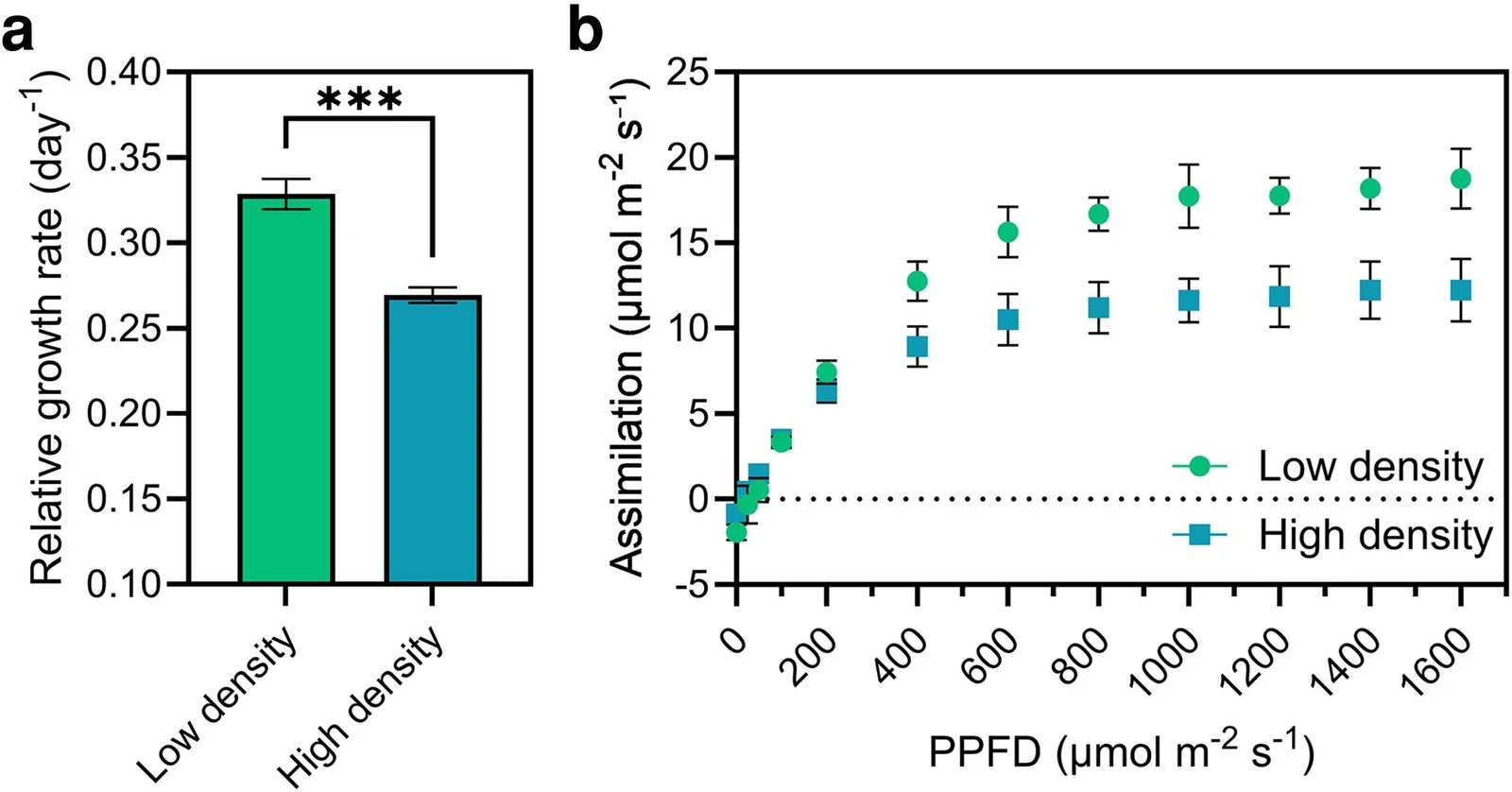
Comparison of low-density-grown (green) and high-density-grown (blue) *W. australiana*. (a) Relative growth rate (day^−1^), Welch's *t*-test performed to test difference between groups (*P* = 0.0002; n = 4, mean ± SD). (b) Assimilation as a function of increasing PPFD (light response curve), normalized to area. A two-way ANOVA determined significant effects of PPFD (*P* < 0.0001), density (*P* = 0.0126), and PPFD × density (*P* = 0.0038; n = 3; mean ± SD).

To assess density effects on photosynthesis, *W*. *australiana* was grown for 10 days at low density (starting with 6 fronds and ending with 160 ± 14 per well) and high density (starting with 36 fronds and ending with 533 ± 23 fronds per well). Low-density-grown duckweeds had a significantly higher mean relative growth rate (0.33 d^−1^ vs 0.27 d^−1^) (Fig. 4a) over this period. During the light response curve, chamber conditions aimed to imitate the crowding experienced in the growth environment, achieved by loading 56.99 mm^2^ (±5.78) for low density and 115.64 mm^2^ (±9.07) for high density duckweed. Low-density-grown duckweeds had higher CO_2_ assimilation above 400 µmol m^−2^ s^−1^ PPFD (Fig. 4b; Figure S3), with the two-way ANOVA identifying a significant interaction between light, density, and light × density.

**Figure 5:**
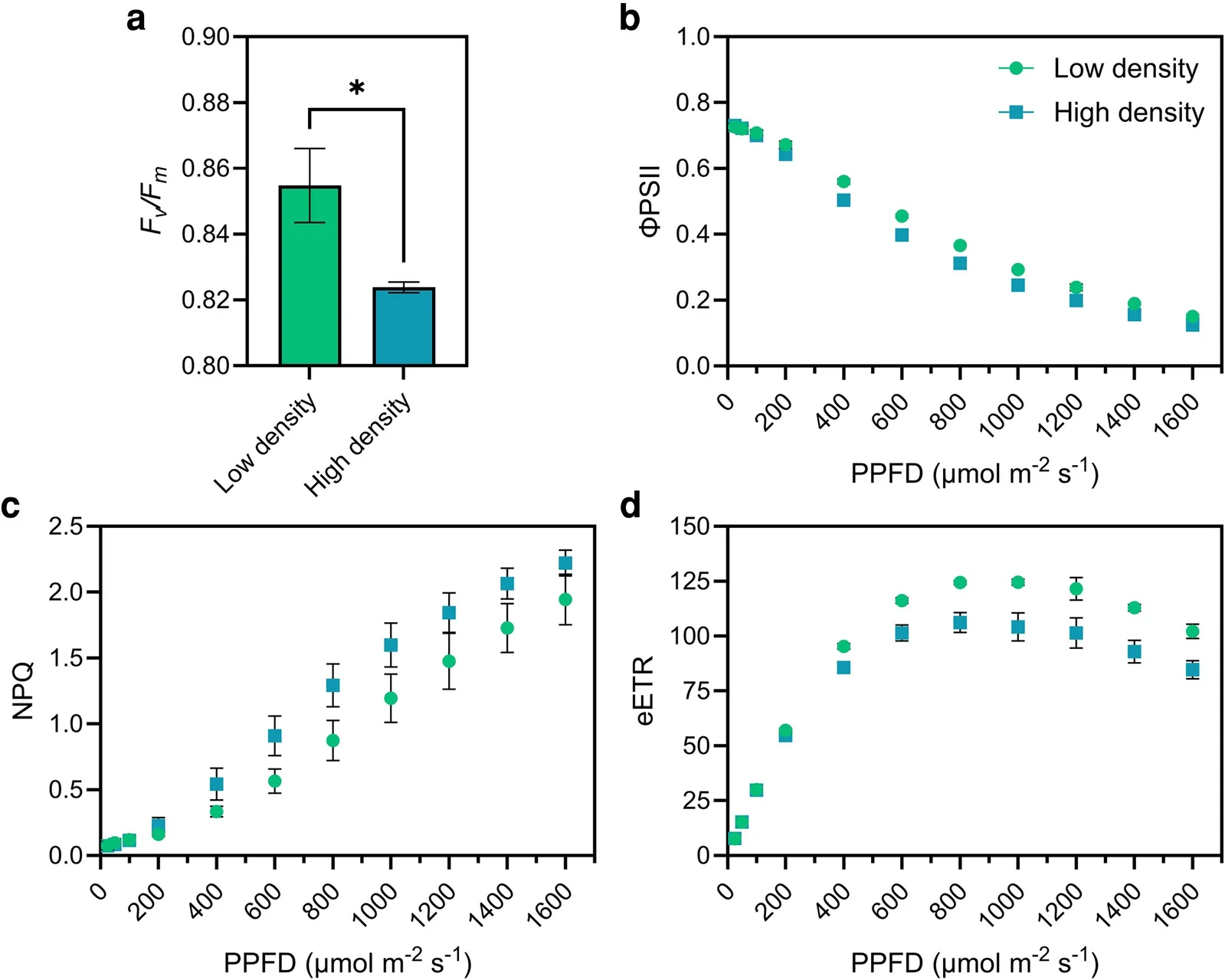
Chlorophyll fluorescence parameters for low (green) and high (blue) density grown *W. australiana*. (a) Maximum operating efficiency of photosystem II (PSII; *F*_v_/*F*_m_), Welch's *t*-test performed to determine a significant difference between groups (*P* = 0.0388). (b) Light-adapted operating efficiency of PSII [ΦPSII; (*F*_m_’-*F*’)/*F*_m_’]. A two-way ANOVA determined significant effects of PPFD (*P* < 0.0001), density (*P* = 0.027), and PPFD × density (*P* = 0.0049). (c) Nonphotochemical quenching [NPQ; (*F*_m_-*F*_m_’)/*F*_m_’]. A two-way ANOVA determined significant effects of PPFD (*P* < 0.0001), density (*P* = 0.0435), and PPFD × density (*P* = 0.0447). (d) Estimated electron transport rate (ΦPSII ⋅ PPFD ⋅ 0.85 ⋅ 0.5). A two-way ANOVA determined significant effects of PPFD (*P* < 0.0001), density (*P* = 0.0033), and PPFD × density (*P* = 0.001), means ± SD (n = 3).

To investigate whether density-dependent differences extended beyond assimilation rates, chlorophyll fluorescence was combined with gas exchange measurements (Fig. 5). Low density duckweed had a significantly higher maximum operating efficiency of photosystem II (PSII; *F*_v_/*F*_m_) (Fig. 5a) and greater operating efficiencies across all investigated light intensities (light-adapted operating efficiency of PSII [ΦPSII]) (Fig. 5b). Nonphotochemical quenching (NPQ) (Fig. 5c) was higher in high-density-grown duckweeds, particularly above a PPFD of 400 µmol m^−2^ s^−1^. Estimated electron transport rate (eETR) (Fig. 5d) peaked between 800 and 1,000 µmol m^−2^ s^−1^ for both densities, before declining at higher PPFD. For all curves, there was both a significant interaction between density and density × PPFD, tested via two-way ANOVA.

Table 2 summarizes light response and chlorophyll fluorescence parameters. While low-density-grown *W. australiana* had a significantly higher *A*_sat_, there were no significant differences between all other light response-derived parameters. At growth PPFD (Q = 100 µmol m^−2^ s^−1^), assimilation, NPQ, and eETR were similar between densities, though low-density-grown duckweeds did have a small but significantly higher ΦPSII. At high PPFD (1,000 µmol m^−2^ s^−1^), low-density-grown *W. australiana* had improved photosynthetic performance, yielding significantly higher ΦPSII and eETR, as well as significantly lower NPQ.

**Table 2:** Summary of low- and high-density-grown *W. australiana* parameters.

Parameters	Low-density	High-density	*P* value
Light response curve			
AQ10_0_, µmol m^−2^ s^−1^	3.3 ± 0.34^a^	3.52 ± 0.28^a^	0.5059
*A*_sat_, µmol m^−2^ s^−1^	21.94 ± 2.33^a^	14.45 ± 1.67^b^	0.0134
LCP, µmol m^−2^ s^−1^	34.31 ± 12.52^a^	16.77 ± 1.33^a^	0.1346
LSP_90%_, µmol m^−2^ s^−1^	1245 ± 343^a^	1464 ± 189^a^	0.4011
*R*_d_, µmol m^−2^ s^−1^	1.86 ± 0.79^a^	0.89 ± 0.12^a^	0.1654
Chlorophyll fluorescence			
*F*_v_ */F*_m_	0.855 ± 0.011^a^	0.824 ± 0.002^b^	0.0388
ΦPSII_Q100_	0.708 ± 0.008^a^	0.701 ± 0.001^b^	0.0365
ΦPSII_Q1000_	0.293 ± 0.003^a^	0.245 ± 0.015^b^	0.0265
NPQ_Q100_	0.12 ± 0.013^a^	0.117 ± 0.009^a^	0.7241
NPQ_Q1000_	1.195 ± 0.183^b^	1.599 ± 0.167^a^	0.0481
eETR_Q100_	30.09 ± 0.342^a^	29.77 ± 0.047^a^	0.242
eETR_Q1000_	124.6 ± 1.442^a^	104.2 ± 6.412^b^	0.0265

Light response curve was fitted using the R *photosynthesis* package (Stinziano et al. 2023). Parameters denoted by _Q100_ and _Q1000_ are measurements at PPFDs of 100 and 1,000 µmol m^−2^ s^−1^, respectively. Different letters indicate significant differences between density treatments according to Welch's t-test (*P* < 0.05). Treatments sharing a letter are not significantly different. Data are means ± SD (n = 3).

Abbreviations: ΦPSII, operating efficiency of photosystem II [(*F*_m_’-*F*’)/*F*_m_’]; AQ10_0_, assimilation at growth light intensity; *A*_sat_, assimilation at saturating PPFD; eETR, estimated electron transport rate (ΦPSII ⋅ PPFD ⋅ 0.85 ⋅ 0.5); *F*_v_/*F*_m_, maximum operating efficiency of photosystem II; LCP, light compensation point; LSP_90%_, 90% of the light saturation point; NPQ, nonphotochemical quenching [(*F*_m_-*F*_m_’)/*F*_m_’]; *R*_d_, dark respiration.

**Figure 6:**
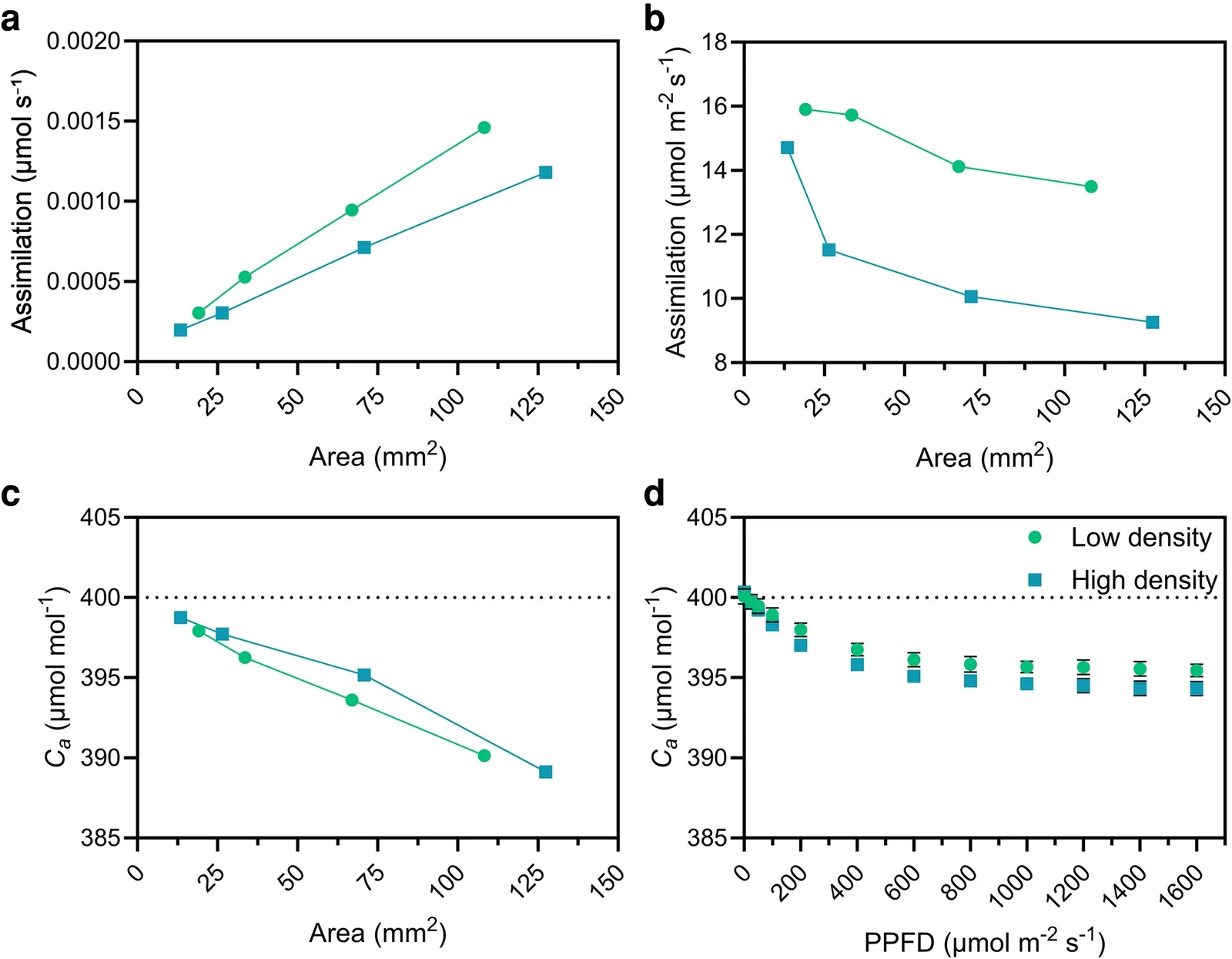
Low- and high-density-grown *W. australiana* assimilation as a function of increasing sample surface area. (a) Non-normalized and (b) normalized to area. Measurements were made at a 400 µmol mol^−1^ CO_2_ reference and 1,000 µmol m^−2^ s^−1^ PPFD (n = 1). Headspace correction of *C_a_* for area response (c) and light response curve (d) (n = 3, mean ± SD), with CO_2_ setpoint indicated by dotted line. Frond area for light response curve: 56.99 mm^2^ (± 5.78) low density and 115.64 mm^2^ (±9.07) high density.

Whilst these parameters indicate differences between *W. australiana* grown at low and high plant densities, it is possible the amount of duckweed loaded into the chamber also influences assimilation. To investigate this, an “area” curve was run. Non-normalized CO_2_ assimilation increases with the surface area of *W. australiana* for both low and high densities; however, the slope for low-density-grown *W. australiana* is steeper, which translates to higher assimilation values when normalized to surface area (Fig. 6a,b). CO_2_ assimilation decreases for both groups with increased surface area loaded into the sample well, with the magnitude of decline being greater for high-density-grown *W. australiana*.

After correcting the reported *C*_a_, it is apparent that both the surface area of the loaded sample (Fig. 6c) and the assimilation rate of the sample (Fig. 6d) influenced the headspace CO_2_ mole fraction. This variation occurs within the range of 10 µmol mol^−1^ with low to high loading (representative loading shown in Figure S4) and within the range of 6 µmol mol^−1^ with low to high assimilation rate. Given the consistency in the variation of the headspace CO_2_ mole fraction from the intended CO_2_ setpoint (in both cases 400 µmol m^−2^ s^−1^), both low- and high-density-grown duckweeds experienced similar corrected headspace conditions, potentially equalized by the high assimilation rates of low-density-grown duckweeds and the highly loaded surface area of high-density-grown duckweeds.

## Discussion

### Duckweed gas exchange methods facilitate investigation of assimilation

Duckweeds are aquatic, floating, fast-growing angiosperms. We report here duckweed carbon assimilation in realistic sample well scenarios in response to light, CO_2_, and temperature. Additionally, we explored how fundamental features of duckweed growth relate to directly measured CO_2_ assimilation and chlorophyll fluorescence. We compared 3 contrasting duckweed species with commercial or environmental significance: *W. australiana*, *S. polyrhiza*, and *L. minuta*. The suite of methodologies discussed here will enable further mechanistic studies into duckweed carbon assimilation, growth, and response to environmental conditions, with relevance for ecology, plant sciences, biotechnology, food and biomanufacturing.

### Limitations in calculating *C*_i_

A key limitation of the aquatic chamber system is the inability to directly measure stomatal conductance (*g*_s_) and, consequently, *C*_i_. In terrestrial leaf measurements, *C*_i_ is calculated from *g*_s_, which is derived from measured water vapor flux between the leaf and atmosphere. However, in the aquatic chamber, water vapor exchange occurs between the media surface and chamber headspace in addition to stomatal transpiration, preventing isolation of the stomatal component of conductance. Alternative calculations of *C*_i_, such as using a custom single-sided chamber (Sharkey et al. 1982), do not work for duckweed.

Without *C*_i_ values, we cannot generate *A/C*_i_ curves, which are pivotal for diagnosing photosynthetic limitations and partitioning control between biochemical capacity and diffusional constraints. While a one-point method exists (De Kauwe et al. 2016), this also requires *C*_i_ and the Michaelis-Menten constant for Rubisco, both yet to be determined in duckweed. However, there is an opportunity to perform a diagnostic test to understand how duckweed responds to key environmental stimuli that we have not been able to do in the past. We therefore attempted to identify the likely upper and lower constraints of *V*_cmax_ and *J*_max_ within duckweed species. To do this, we present *A/C*_a_ curves estimating *V*_cmax_ and *J*_max_ by assuming *C*_i_ represents a fixed proportion of *C*_a_ (Figure S5). This introduces uncertainty into parameter estimates, as evidenced by the wide ranges obtained when assuming *C*_i_ at 100%, 66%, and 33% of *C*_a_ (Table 1). Future methodological development includes alternative approaches for estimating *C*_i_ in aquatic systems, such as stable isotope discrimination techniques.

### Photosynthetic insights

*A*_sat_ is typically between 10 and 20 µmol m^−2^ s^−1^ for many C_3_ species (Busch et al. 2024), with the measured 3 duckweed species lying within or close to this range (Table 1). *A*_opt_ is typically between 10 and 30 µmol m^−2^ s^−1^ (Busch et al. 2024), and again, the duckweed species were close to this range. For *L. minuta*, however, *A*_opt_ was lower than typical at 7.61 µmol m^−2^ s^−1^, suggesting a reduced maximum photosynthetic capacity. A lower *A*_opt_ reflects limited Rubisco carboxylation or electron transport (Yamori et al. 2010), which is reflected in the lower *V*_cmax_ and *J*_max_ values for *L. minuta*, compared with both other duckweed species. Typical higher plant values range between 50 and 150 µmol m^−2^ s^−1^ for *V*_cmax_ and around 200 µmol m^−2^ s^−1^ for *J*_max_ (Busch et al. 2024). Assuming that *C*_i_ was 100%, 66%, and 33% of *C*_a_, the *V*_cmax_ ranged from 59.79 to 136.9 µmol m^−2^ s^−1^, 29.62 to 66.32 µmol m^−2^ s^−1^, and 22.50 to 51.46 µmol m^−2^ s^−1^ for *W. australiana*, *S. polyrhiza*, and *L. minuta*, respectively (Table 1).

### Environmental insights

The methods we developed allow us to probe the environmental responses of duckweed and determine optimal conditions for growth in controlled environments. If growing all 3 species together, predicted optimal conditions for assimilation would be a PPFD of 1,000 µmol m^−2^ s^−1^, CO_2_ concentration of 700 µmol mol^−1^, and temperature of 35 °C (Table 1). It remains to be determined if these short-term point measures translated into better growth over sustained periods. *W. australiana* is particularly responsive to increased CO_2_, compared with *S. polyrhiza* and *L. minuta*. Also, *L. minuta* had the least variable responses to different temperatures but had generally low CO_2_ assimilation (Fig. 1).

### Duckweed normalization

We presented 4 methods for duckweed normalization: area, weight (fresh and dry), and chlorophyll content (see Methods and Results). Area normalization is the standard for leaves because of the close relationship between photosynthetic rate, light interception/absorbance, and plant growth rate. Instrumentation has been adapted accordingly, with leaves either filling the sample well or with area easily measured non-destructively. For duckweeds, the same principle follows, with surface area commonly used to measure growth rate due to new fronds spreading across the media surface. When considering surface area-normalized assimilation, *W. australiana* consistently outperformed *S. polyrhiza* and *L. minuta*. With *W. australiana* species generally linked to higher growth rates, this higher assimilation rate supports their source-sink relationship where rootless duckweeds have less “superfluous” biomass than rooted duckweeds and are therefore able to asexually reproduce more vigorously (Stewart et al. 2021; Ziegler et al. 2023). Furthermore, as stomatal density did not differ significantly between the 3 duckweed species (Table S1), the consistently higher assimilation in *W. australiana* cannot be attributed to greater stomatal number and instead indicates greater intrinsic photosynthetic capacity.

Chlorophyll content per unit leaf (or frond) area is commonly used as an indication of the investment of resources such as nitrogen into photosynthetic components and, as such, can denote Rubisco-limited photosynthetic capacity. This applies under high and often excessive light conditions where chlorophyll content per se does not limit photosynthetic rate. Under low light, a higher chlorophyll content can arise from preferential investment into light harvesting complexes to enable greater light capture ability alongside other low-light adaptations such as reduced respiration. However, excessive pigmentation creates self-shading problems: recent work with crop plants has identified a reduced chlorophyll content as being beneficial (Walker et al. 2018). Therefore, a reduced photosynthetic rate per unit chlorophyll might indicate an overinvestment for the specific light conditions. We therefore determined the chlorophyll content of each species and estimated total chlorophyll on an area basis for each sample. *W. australiana* has both the highest assimilation rates per unit of chlorophyll and chlorophyll content, which corresponds to the high *V*_cmax_ and *J*_max_, compared with the more modest assimilation and chlorophyll content of *S. polyrhiza* (Fig. 2; Table 1). This suggests that the denser chlorophyll content of *W. australiana* is an indicator of higher investment into photosynthetic components, especially Rubisco, helping to drive the greater carbon assimilation.

However, *W. australiana* assimilation is markedly lower than the Lemnoideae species when normalized by fresh weight (Fig. 2). While there could be a number of potential explanations, including higher starch content, we also point out differences in morphology, where *S. polyrhiza* and *L. minuta* species have been shown to have more air spaces than *Wolffiella lingulata* (Jones et al. 2021). This correlates with our observation that *W. australiana* fronds were “denser” when comparing the corresponding weights for areas loaded into the sample wells.

### Diurnal assimilation

Assimilation was consistent throughout the day in *S. polyrhiza* (Fig. 3), at least after initial ramping when lights were first turned on, possibly caused by slow induction of Calvin cycle enzymes. Within 1 h, assimilation reached a steady state and did not significantly change for the rest of the day. Higher plants retain circadian clock entrainment of stomata that causes a characteristic bell-shaped, unimodal curve in conductance with a clear maximum around the middle of the day. In higher plants where stomata exert some control over photosynthesis, both conductance and CO_2_ assimilation rates can show this pattern (Hennessey and Field 1991). However, under benign, humid, low-light-controlled environment conditions, there is less stomatal control even for plants like Arabidopsis, and the bell curve is much less clear for CO_2_ assimilation (Dodd et al. 2005). Nonetheless, our data are consistent with the suggestion that duckweeds keep stomata fully open and hence do not exert active regulation over photosynthesis (Michael et al. 2021; Stewart et al. 2021).

### Growth density

Exploring the effects of different plant growth densities and sample well densities helped to answer several questions about the technical accuracy of data and the biology of duckweed. First, reliable chlorophyll fluorescence signals were obtained even at the lowest sample well densities. Also, gas equilibration times were manageable across different densities. A bigger technical question arose with the reduced headspace in the sample well at higher densities, suggesting that similar densities should be used to avoid problems. However, the lower [CO_2_] values at higher densities (Fig. 6c) can be compared to the *A*/*C*_a_ response curves, and here, the drop from 398 to 388 ppm CO_2_ across the whole range of densities results in a difference of around 1 µmol CO_2_ m^−2^ s^−1^.

In a similar way that aging of leaves (senescence) results in a decline in photosynthetic rate, it is necessary to understand whether photosynthetic rates depend on meticulously maintained young, fresh stocks or if denser duckweeds assimilate CO_2_ as effectively. Denser stocks had markedly reduced photosynthetic capacities per unit area. Individual frond age could be a contributory factor, but this has proven elusive to measure, and duckweed lifecycle stage likely contributes to variation within samples irrespective of density. Aside from an age-dependent factor, acclimation to density may involve an environmental response where individual fronds within crowded areas are more conservative with resources. The use of the aquatic chamber therefore requires users to be systematic in monitoring growth rate, density, and chronological age of samples and carefully balance stock and sample well density, as assimilation is sensitive to both.

A separate question arises as to whether density within the sample well influences frond photosynthetic capacity during the measurement. From the results presented, a sample well density-dependent effect was apparent in both low- and high-density-grown duckweeds. While including more duckweed fronds in the sample well increased assimilation in a linear manner, the magnitude of the decline in assimilation rates per unit frond area was difficult to account for simply from the magnitude of the reduction in CO_2_ caused by the loss of headspace. This is an intriguing outcome possibly caused by technical issues (further complications of headspace, issues of light interception) or an unknown biological interaction between fronds that downregulates photosynthesis rapidly in denser cultures.

### Further work

The gas exchange methodology and best practices described here enable mechanistic studies of duckweed photosynthesis across multiple scales and applications. For physiological experiments, these methods allow investigation of how different species or clones respond to environmental stresses, including nutrient limitation, heavy metal exposure, extreme temperatures, and high light intensity. The ability to combine gas exchange with chlorophyll fluorescence provides insight into both carbon assimilation and the underlying photochemistry, enabling researchers to diagnose specific limitations and stress responses.

From an ecological perspective, duckweeds are globally distributed across nearly every continent (Smith et al. 2025) and contribute significantly to carbon cycling in freshwater ecosystems. The methods developed here could be applied to field-collected duckweed populations to understand local adaptation, phenotypic plasticity, and responses to environmental gradients. Such studies could inform models predicting how duckweed populations will respond to climate change, particularly warming temperatures and altered precipitation patterns.

For biotechnology applications, optimization of photosynthetic performance is key to maximizing biomass production for biomanufacturing, biofuel production, and protein-rich food sources (Ofoedu et al. 2025). The ability to rapidly screen duckweed lines for photosynthetic performance under controlled conditions and to identify optimal growth conditions for specific applications represents a significant advance. Recent development of transformation protocols for *S. polyrhiza* (Barragán-Borrero et al. 2025) raises the possibility of genetically engineering enhanced photosynthetic traits, which could be screened using these gas exchange methods.

To further investigate density-dependent effects, future work should examine duckweeds sampled from different positions within larger growth containers, comparing fronds at the edge of a colony (where they experience less crowding) to those in the center (where they are surrounded by neighboring fronds). This would better mimic natural pond conditions and could reveal whether the density response we observed represents an adaptive strategy for resource allocation in crowded environments. Duckweeds also present unique opportunities as a model system for studying photosynthetic variation across asexual generations. Because they reproduce clonally (Ho et al. 2019), it may be possible to track how photosynthetic capacity changes with frond age and generation number within a genetically uniform population. This could reveal insights into aging effects within a population or epigenetic regulation of photosynthesis.

Beyond duckweed, the aquatic chamber system offers promise for understanding gas exchange in other floating aquatic plants. Duckweeds are particularly interesting from an evolutionary perspective as they represent a return to aquatic life after their ancestors became terrestrial (Ware et al. 2023). This evolutionary transition may have left traces in their photosynthetic physiology. Comparative studies with other aquatic plants, including those that have independently returned to water (such as Azolla and Pistia) (Ware et al. 2023) and those that remained aquatic (such as submerged macrophytes), could reveal how photosynthetic systems adapt to the aquatic environment and whether convergent solutions have evolved.

## Conclusions

We developed methods for measuring duckweed photosynthetic gas exchange using an aquatic chamber, revealing species-dependent responses to light, CO_2_, and temperature, with *W. australiana* demonstrating the highest assimilation rates, while *S. polyrhiza* and *L. minuta* had lower photosynthetic capacity. Plant density significantly influenced photosynthetic performance, with implications for cultivation strategies at scale. These best-practice methods enable mechanistic investigations of duckweed photosynthesis, supporting applications in biotechnology, agriculture, and ecology.

## Materials and methods

### Duckweed cultivation

Duckweed clones #44 (*W. australiana*), #53 (*S. polyrhiza*), and #8 (*L. minuta*) were obtained from the Australian Collection of Duckweed Clones (Mortimer and Gilliham labs, Adelaide University, Australia). Cultures were grown in controlled-environment plant growth chambers (Gen 2000, Conviron, Australia), as follows: 25 °C (day and night), 70% relative humidity, 100 µmol m^−2^ s^−1^ PPFD, 16/8 h (day/night), using 6-well plates filled with 5 mL of N-Medium [0.15 mM KH_2_PO_4_, 1 mM Ca(NO_3_)_2_·4H_2_O, 8 mM KNO_3_, 5μM H_3_BO_3_, 13 μM MnCl_2_·4H_2_O, 0.4μM Na_2_MoO_4_·2H_2_O, 1 mM MgSO_4_·7H_2_O, 25 μM FeNaEDTA, pH 5.8] (Appenroth et al. 1996; Appenroth 2023). Cultures were maintained by transferring a single plant (mother frond plus any attached daughter fronds) to a fresh 6-well plate every 7 days. In addition to continuously cultivating healthy biomass for gas exchange measurements, this ensured that density-dependent growth effects would not influence gas exchange measurements.

For comparison of CO_2_ assimilation of duckweeds grown at low and high plant density, the same cultivation method as previously described was used; however, duckweeds grown at low density had an initial 6 fronds transferred into 6-well plate wells, whereas high-density-grown duckweeds had 36 fronds transferred. These were then grown for 10 days, with media replenished in both treatments at day 7. Relative growth rate (RGR) of the duckweed under these treatments was determined as follows:


$$RGR=\frac{\ln(area_{10})\text{-}ln(area_{0})}{t_{10}-t_{0}}$$

### Instrumentation

**Figure 7:**
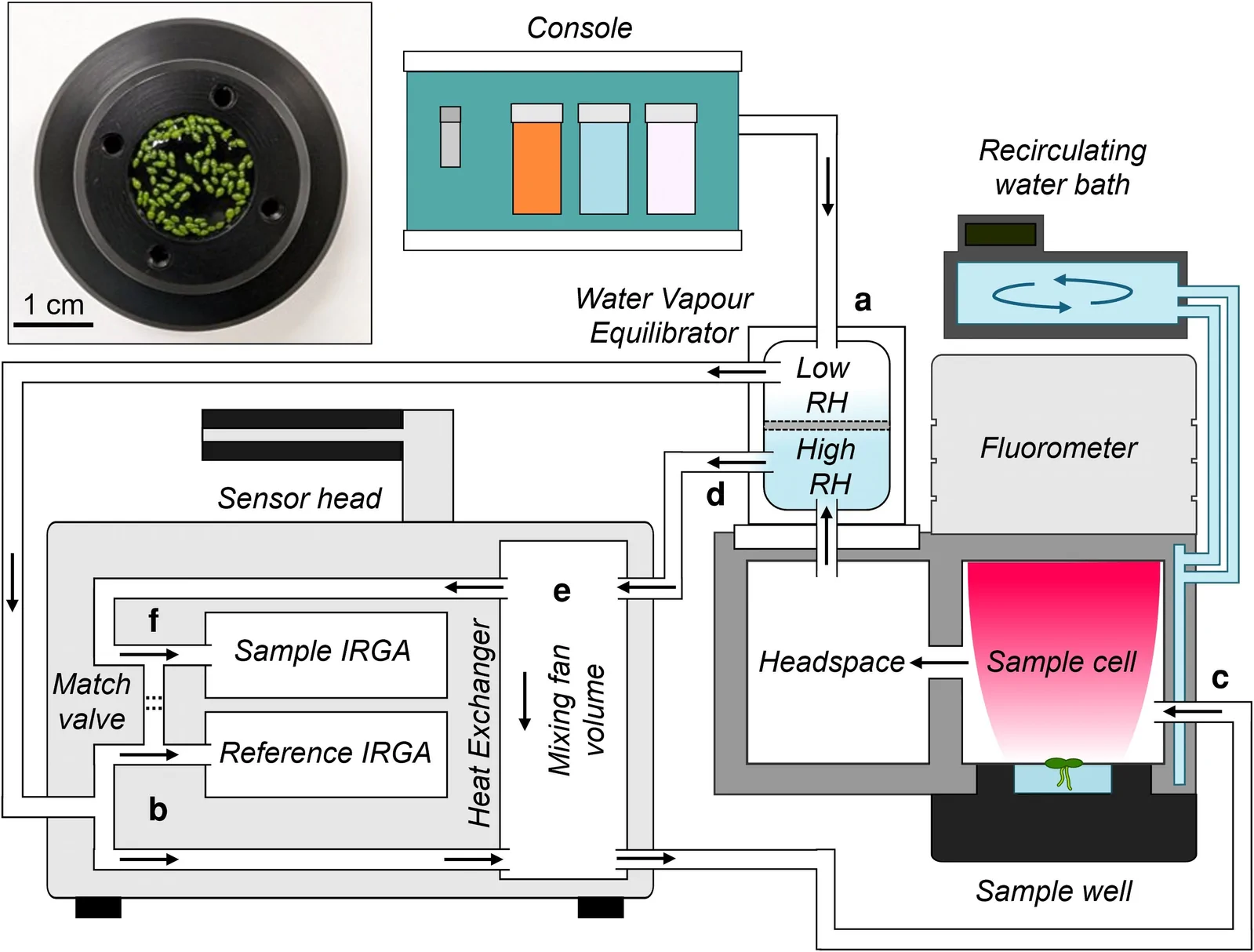
Schematic of the 6800-18 orientation with sample adapter well and airflow diagram. (a) Air from the console enters the water vapor equilibrator and passes through the low-humidity compartment and along to the sensor head. (b) Air is split between flow to the reference infrared gas analysis (IFGA) and along to the mixing fan volume and through to the aquatic chamber sample cell. (c) The duckweed is held in a sample well (top-down view shown in upper left) and is illuminated by the fluorometer light source. The sample cell is a partially jacketed vessel and can be paired with a recirculating water bath for more accurate temperature control. Air passes through the sample cell, into the headspace, and back into the water vapor equilibrator. (d) The water vapor equilibrator is partitioned by a selectively permeable membrane; (e) Sample cell air then passes to the mixing fan volume. The air temperature of this volume is controlled by the sensor head heat exchanger. Airflow between (e), (c), and (d) consists of the subsample loop. (f) For measurement, approximately 30% of this airflow is passed to the sample IRGA.

A full protocol (Gill et al. 2026) is available at dx.doi.org/10.17504/protocols.io.81wgbo111lpk/v1. An LI-6800 portable photosynthesis system (LI-COR, USA) (LI-COR 2022a), equipped with an aquatic chamber (6800-18, LI-COR) (LI-COR 2022b), was used to measure aquatic gas exchange. As built, the aquatic chamber volume can contain an algal suspension, where air is “bubbled” uniformly through the bottom of the chamber, allowing gas exchange between the air and suspension, before flowing through to the sample IRGA for measurement. The water vapor equilibrator minimizes the difference between the reference air and sample air humidity, reducing the humidity delta to a small enough magnitude (<0.05 mmol mol^−1^ in this work) to measure the sample carbon flux. For the duckweed experiments, a sample adapter kit (LI-COR part no. 9968-338) replaced the sample window, intended to allow the user to anchor corals or seaweed and facilitate a larger sample size that does not dislodge from the flow within the chamber. This plug conveniently contains a well that can hold 1 mL of media alongside duckweed samples. By orienting the LI-COR 6800 sensor head and chamber “handle up,” duckweeds are positioned parallel to the airflow, similar to wind blowing across the surface of a pond, with the duckweed covering the media surface (Fig. 7). This allowed duckweeds to be measured closer to in situ gas exchange, in contrast to being bubbled aggressively like algal suspensions.

To increase the accuracy of temperature control, a recirculating water bath was attached to ports on the aquatic chamber. The chamber block is a partially jacketed vessel, allowing a known temperature of water to be circulated, helping to more tightly regulate T_aquatic_, a variable not directly controllable but measured by a thermistor embedded within the sample block. Alongside the control of T_exchange_, which heats air within the mixing fan volume, the recirculating water bath minimized any potential cooling effect from the sample chamber block and ambient air temperature. Thus, the sample temperature was not directly controllable in situ.

For all measurements, unless explicitly stated, flow was 500 µmol s^−1^ with pump speed at auto, relative humidity (air) 70%, CO_2_ (reference; *C*_a_) 400 µmol mol^−1^, temperature (T_exchange_) 25˚C, and mixing fan speed 10,000 rpm.

### Preparing the aquatic chamber for samples

The aquatic chamber must be prepared in 3 steps for aquatic samples: (i) warm-up tests, (ii) hydrating the membrane, and (iii) loading the duckweed samples. Warm-up tests were run with the aquatic pump switched to off (if left on, the sample cell calibration test failed) with no media in the sample adapter well. The sample adapter well was loaded with 1 mL of N-Medium to hydrate the membrane, and the instrument was run for approximately 1 h to sufficiently warm up before loading duckweed samples (Gill et al. 2026).

### Loading duckweed samples

Next, duckweed samples were loaded into the sample adapter well. The 1 mL of N-Medium used to hydrate the membrane from the sample adapter well was replaced. Using an inoculating loop, duckweed fronds were transferred into the sample adapter well; approximately 50 to 70 mm^2^ of duckweed per sample. This roughly equated to the following frond numbers: *W. australiana*, 90 to 110 fronds; *S. polyrhiza*, 4 fronds; and *L. minuta*, 30 fronds. The duckweed and media were allowed to reach steady-state gas exchange before beginning measurements. For conditions similar to stock growth conditions, steady state was reached rapidly, within 2 min. For conditions with much greater CO_2_ or PPFD than ambient, steady state took up to 15 min. No substantial evaporation was observed in the sample adapter well, with >0.9 mL remaining after 3 h of measurements, which is greater than the time for each of the 3 response curves.

### Correcting for incident light on sample plane

A key point of difference to measuring leaf gas exchange in the regular LI-6800 chambers is that incident light on the sample (Q_sample_) in the aquatic chamber is just over 50% of the setpoint (Q_in_). This is due to light loss between the internal surfaces of the chamber and should be corrected (Gill et al. 2026). Set points used for subsequent light responses are shown in Table S1.

### Media optimization and correcting headspace CO_2_

The aquatic chamber has unique constraints that warrant consideration in addition to the best practices usually adopted for leaf gas exchange measurements. First, the equilibration and stability of the media in the chamber must be considered. We show that the N-Medium used for these experiments does not meaningfully alter light or CO_2_ responses (Figure S6). This is of particular importance as duckweeds do not make use of dissolved carbon dioxide; thus, the sorption of CO_2_ into the N-Medium has great potential to influence assimilation values. Key to this is the assumption that gaseous CO_2_ existing within the headspace is in equilibrium with its 2 forms within the media volume: dissolved carbon dioxide and carbonic acid. For a changing CO_2_ concentration (eg, during CO_2_ response curves), we tested N-Medium alone to determine the time required for equilibration, with a minimum wait time of 10 min, allowing both equilibration of the media and steady state for the duckweed CO_2_ assimilation. Matching the reference and sample IRGAs is vital whenever CO_2_ concentration changes, which also acts as a zeroing step.

It should also be noted that measured CO_2_ does not truly reflect the headspace CO_2_ mole fraction as the aquatic chamber is a 2-compartment system, where the sample is in one compartment and the sample IRGA is in the second compartment. Connecting these is the subsample loop, which circulates airflow from the mixing fan volume through the sample chamber and then to either the sample IRGA or back to the sample chamber. As the measured air is only a fraction of the air sampled from the headspace, as opposed to the full volume of headspace air, best practice is to correct for differences in CO_2_ that arise between the 2 compartments (Gill et al. 2026). When we measured the subsample flow rate, we found that around 30% of the flow was directed to the sample IRGA, with the other 70% recirculated into the sample chamber alongside air from the console.

Correction then informs the selection of the CO_2_ setpoints so that the headspace CO_2_ accurately reflects the chosen concentration for measurements. During testing, N-Medium alone was used to determine CO_2_ setpoints for each response curve, where the difference between reference CO_2_ and headspace CO_2_ varied by less than 3 µmol mol^−1^ (Figure S6d). For analysis of *A/C*_a_ curves, headspace CO_2_ was used. It is apparent that when loaded, headspace CO_2_ is sensitive to the assimilation rate of the loaded sample, induced by high PPFD or CO_2_ mole fraction or through increased frond number. However, at most, this varied by around 10 µmol mol^−1^ CO_2_ and, for most curves, was much less (Figure S7).

### Light response curves

Light response curves (*A/I*_inc_) are used to measure CO_2_ assimilation as a function of increasing PPFD. Here, the response of duckweed to changing light intensities was probed, and the light-saturated CO_2_ assimilation rate at ambient CO_2_ (A_sat_) (Busch et al. 2024) was determined. Light response curves were run close to ambient CO_2_, at 400 µmol mol^−1^, with duckweed equilibrated in the chamber at high PPFD (2,979.52 µmol m^−2^ s^−1^) until it reached steady state (10–15 min) (Gill et al. 2026). The light response program was run at 13 stepwise decreases of PPFD at Q_sample_ (µmol m^−2^ s^−1^): 1,600, 1,400, 1,200, 1,000, 800, 600, 400, 200, 100, 50, and 0. During measurement, the minimum and maximum wait times were set at 120 and 180 s, respectively, with a total maximum runtime of 39 min.

### CO_2_ response curves

In routine photosynthetic analysis, CO_2_ assimilation as a function of increasing intercellular CO_2_ concentrations (*A/C*_i_ curves) is used to estimate a range of biochemical parameters from the Farquhar et al. model (Farquhar et al. 1980; Busch et al. 2024). However, it is not possible to measure *C*_i_ in the aquatic chamber. Therefore, CO_2_ assimilation as a function of increasing reference CO_2_ concentration (*C*_a_) is presented instead. The aim of the CO_2_ response curves was to understand the response of duckweed to CO_2_ and to calculate key parameters including the maximum rate of Rubisco carboxylation (*V*_cmax_) and maximum rate of electron transport (*J*_max_). PPFD was chosen based on the previously measured light response curve for each species, with 1,000 µmol m^−2^ s^−1^ at the sample (Q_in_ of 1,862.20 µmol m^−2^ s^−1^) deemed saturating for all 3 species. The CO_2_ response program was run using 14 CO_2_ reference concentration (µmol mol^−1^) setpoints: 400, 300, 200, 100, 50, 0, 400, 500, 600, 700, 800, 1,000, 1,200, and 1,400 (Gill et al. 2026). The minimum and maximum wait times were 600 and 660 s, respectively, with a total maximum runtime of 2 h 34 min. It is important that the wait time is not too short to allow the sample to adjust to the new CO_2_ concentration, but also not too long, as the biochemistry of the sample will be altered over time. Matching was vital at every CO_2_ concentration to ensure that the reference and sample analyzers were close to equal.

### Temperature response curves

Curves showing CO_2_ assimilation as a function of increasing temperature (temperature response curves [*A/T*]) were used to estimate optimum photosynthetic temperature (*T*_opt_) and the associated CO_2_ assimilation rate (*A*_opt_). The aim here was to understand the response of duckweeds to temperature at a saturating PPFD and ambient CO_2_ concentration. The PPFD setpoints were chosen using the previously determined saturating PPFD (ie, 1,000 µmol m^−2^ s^−1^ at the sample [Q_in_ of 1,862.20 µmol m^−2^ s^−1^]), alongside a close-to-ambient CO_2_ concentration of 400 µmol mol^−1^ CO_2_. A recirculating water bath was used to control the temperature of T_aquatic_, starting with the lowest temperature setpoint, with T_exchange_ set to the same temperature. The temperatures were as follows (°C): 17.5, 20, 22.5, 25, 27.5, 30, 32.5, 35, and 37.5. The water bath took approximately 15 min to reach each temperature. Once T_aquatic_ stabilized to the same temperature as the water bath, samples were held at the respective temperature for 300 s before logging. To reduce variability, an average of the CO_2_ assimilation over the previous 45 s was logged. The total runtime for these curves was about 3 h.

### Instantaneous measurements

Instantaneous measurements of CO_2_ assimilation were taken over a 16-h diurnal period. In this instance, environmental conditions were set to 1,000 µmol m^−2^ s^−1^ at the sample (Q_in_ of 1,862.2 µmol m^−2^ s^−1^), 25˚C, and a relative humidity of 70%. Samples were loaded into the chamber before the start of the light period, with measurements taken every 5 min from just before lights on at 7:00 AM to lights off at 11:00 PM. The CO_2_ canister was replaced at 3:00 PM (midpoint, 8 h), with the N-Medium in the sample adapter well also replenished at 3:00 PM, as it had evaporated to approximately half (0.5 mL).

### Fluorescence measurements

The LI-6800 fluorometer was used to resolve photosynthetic parameters of interest: Maximum operating efficiency of photosystem II (*F*_v_/*F*_m_), light-adapted operating efficiency of PSII [ΦPSII; (*F*_m_’-*F*’)/*F*_m_’], and NPQ, [(*F*_m_-*F*_m_’)/*F*_m_’]. An estimated ETR (eETR) was calculated as


eETR=ΦPSII⋅PPFD⋅0.85⋅0.5

where 0.85 is the assumed absorbance for green leaf material and 0.5 accounts for the energy split between photosystem I and PSII. These parameters can be determined using a light response protocol, involving a stepwise increase in PPFD paired with saturating pulses to generate data on PSII activity after an initial dark adaptation period of 30 min.

Duckweeds were loaded into the sample well and dark-adapted in situ, with measurements taken at the following setpoints (PPFD, µmol m^−2^ s^−1^): 0, 25, 50, 100, 200, 400, 600, 800, 1,000, 1,200, 1,400, and 1,600. Gas exchange measurements were taken alongside the fluorescence measurements using 5-min wait times. When combining gas exchange and fluorescence, LI-COR recommends limiting the sample blue light to 40 µmol m^−2^ s^−1^. This requires recalculation of Q_in_ set points due to the higher fraction of red light transmitting through the sample cell, resulting in a slightly lower Q_in_ (Table S2).

### Sample normalization

With the aquatic chamber, CO_2_ assimilation is reported in µmol s^−1^ and therefore must be normalized to allow comparison between samples; this is done by dividing the raw assimilation by the desired unit. Outlined are 4 approaches to normalizing raw assimilation data. Normalizing assimilation data to surface area (µmol m^−2^ s^−1^) is the most common and easily comparable to leaf gas exchange measurements. For duckweeds, however, it may be appropriate to normalize by fresh weight (µmol g^−1^ s^−1^) to account for varying morphology in photosynthetic tissue below the media surface. Considering the substantial water weight of duckweeds, dry weight (µmol g^−1^ s^−1^) is also presented, alongside total chlorophyll content (µmol unit chlorophyll^−1^ s^−1^) given its role in light capture.

Area normalization was carried out prior to loading samples. A ruler was placed alongside the sample well on the same plane as the duckweeds, and an image was taken from an aerial view. Duckweed area was measured using ImageJ (Schneider et al. 2012). The image scale was set using the ruler and the 2D surface area was determined using the color threshold tool, with parameters corresponding to the green area of duckweed fronds. Given the curved nature of fronds, truly accurate determination of surface area is difficult; however, this method is a practical estimate of area.

For fresh weight normalization, sample weight was measured after gas exchange measurements. Using a spray bottle or inoculation loop, duckweeds were gently dislodged from the sample well into a cell strainer, which was blotted dry thoroughly. Duckweed fresh weight was then measured using a fine balance.

For dry weight normalization, duckweeds from the same 6-well plates as those used for gas exchange measurements were flash-frozen in liquid nitrogen, then freeze-dried for 16 h.

Using the same freeze-dried samples, total chlorophyll a + b content was determined as described in Lichtenthaler and Babani (2022). For duckweed, 50 mg was ground in 1 mL 80% acetone using 3 mm ceramic beads and a tissue homogenizer (SPEX SamplePrep 2010 Geno/Grinder, USA). The samples were stored overnight at −20˚C to allow full extraction of chlorophyll from tissue. Samples were centrifuged at 2,900 × *g* for 10 min; the supernatant was aliquoted off and centrifuged again at 2,900 × *g* for 10 min. This supernatant was then measured using a spectrophotometer (Biotek µQuant MQX200 Microplate Spectrophotometer, Australia) at 662 and 645 nm wavelengths. Total chlorophyll content of the sample (µg mL^−1^) was calculated as follows:


$$\mathrm{Total}\;\mathrm{chlorophyll}=(11.24A_{662}\text{–}2.04A_{645})+(20.13A_{645}\text{–}4.19A_{662})$$

Total chlorophyll was then corrected according to the dilution factor and normalized to the initial area of the fresh duckweed prior to freeze-drying to give milligrams of chlorophyll per m^−2^ of frond for each species. This allowed estimation of chlorophyll content loaded into the sample well for each replicate.

### Stomatal density

Stomatal density was measured in all 3 species using an inverted microscope (Nikon Diaphot 200) at 20× magnification. Six non-overlapping images of the adaxial surface were captured per biological replicate (n = 3), each corresponding to a calibrated area of 0.08165 mm^2^. Stomata were counted manually from captured images and stomatal density calculated as stomata mm^−2^.

### Data analysis and curve fitting

Once log files were downloaded from the LI-6800, an R script was used to correct the headspace CO_2_, and the incident light on the sample, and assimilation was normalized to area, weight, and chlorophyll content (Dataset S1). Curves and subsequent figures were plotted in GraphPad Prism (v. 10.5.0) and fitted using the R *photosynthesis* package (v. 2.1.5) (Stinziano et al. 2021, 2023) within RStudio (2025.09.0 + 387) (Dataset S2). Species differences between curve fitted parameters were tested using an ordinary one-way ANOVA, followed by Tukey's multiple comparisons tests, with α of 0.05 and compact letter summary generated using *multcompView* package (Piepho 2012). GraphPad Prism was also used to test relative growth rates (Welch's *t*-test, α 0.05) and chlorophyll fluorescence data (two-way ANOVA testing plant density and equipment for each parameter, Tukey corrected for multiple comparisons, α 0.05).

## Supplementary Material

kiag311_Supplementary_Data

## Data Availability

The data underlying this article are available in the supplementary materials.
