# Supplementary material for: Developing carbon assimilation methods in duckweed for insights into photosynthesis and growth mechanisms
Source: Plant Physiol. 2026 May 26;202(1):kiag311. doi: 10.1093/plphys/kiag311 (PMC13544436; doi:10.1093/plphys/kiag311)
Supplement: Supplementary file 1 [file kiag311_supplementary_data.zip › Duckweed methods Supplementary Data.pdf]

Supplementary Material

Developing carbon assimilation methods in duckweed for new insights into photosynthesis and growth mechanisms

Robert C. Rintoul*, Alison R. Gill*, Lorna McAusland, Erik H. Murchie, Matthew Gilliham, Jenny C. Mortimer (* joint first authors)

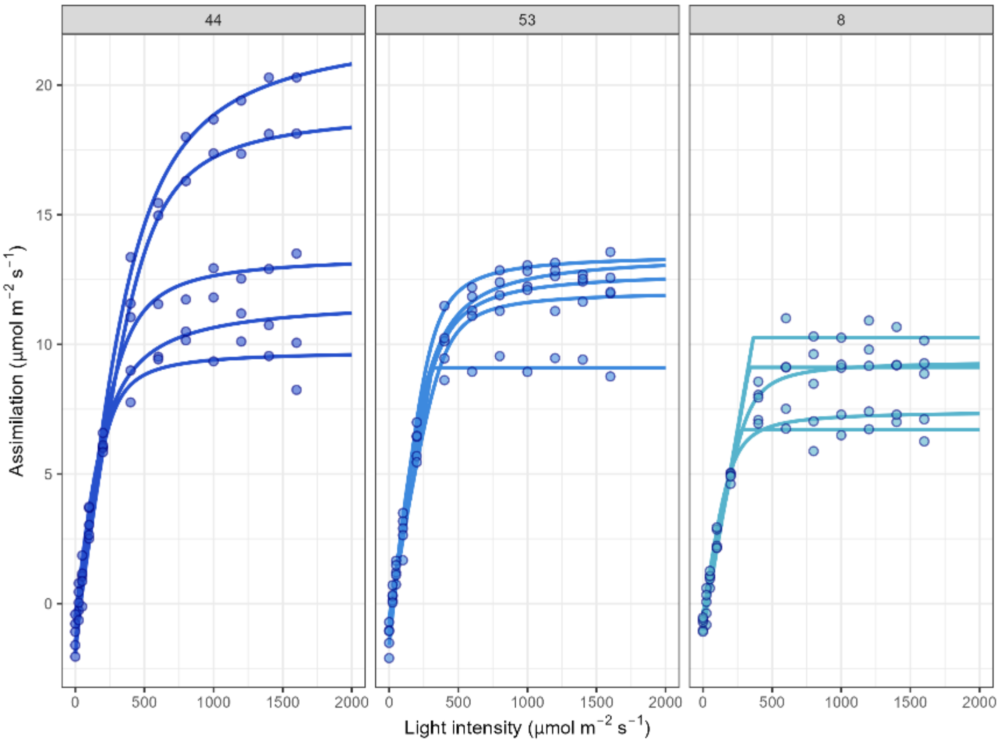

Supplementary Figure S1. Light response curves (*A/Q*) fitted by the *photosynthesis* package in R, for three duckweed species (44 = *Wolffia australiana*, 53 = *Spirodela polyrhiza*, 8 = *Lemna minuta*). Points represent individual measurements and solid lines represent modelled *A/Q* curves fitted to each replicate. $n = 5$.

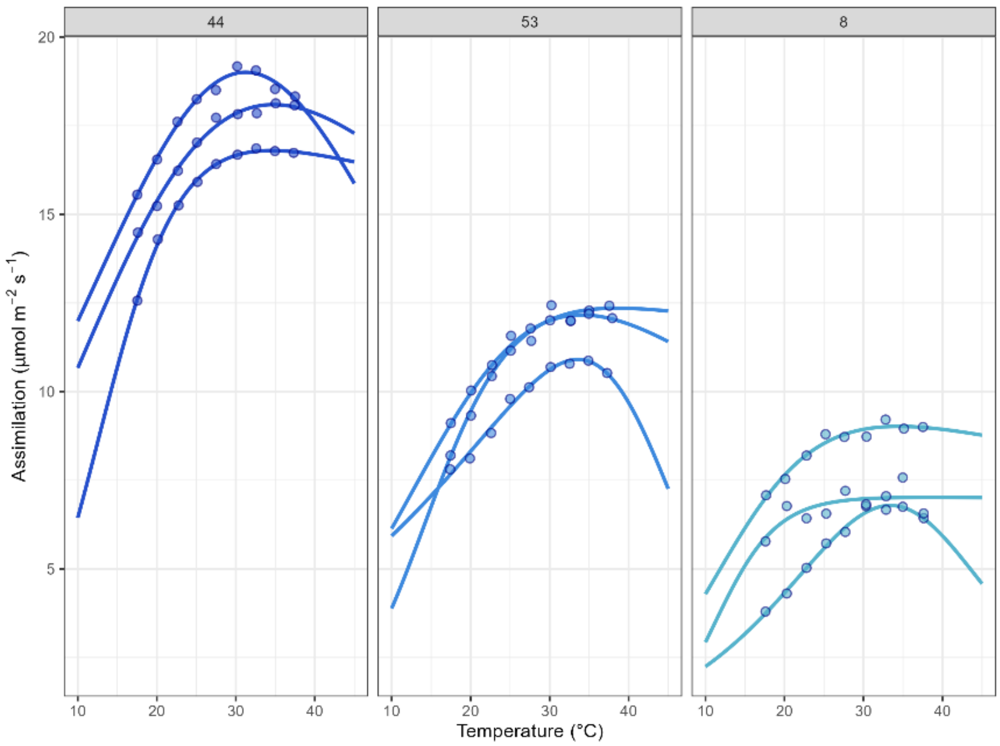

Supplementary Figure S2. Temperature response curves (*A/T)* fitted by the *photosynthesis* package in R, for three duckweed species (44 = *Wolffia australiana*, 53 = *Spirodela polyrhiza*, 8 = *Lemna minuta*). Points represent individual measurements and solid lines represent modelled curves fitted to each replicate. n = 3.

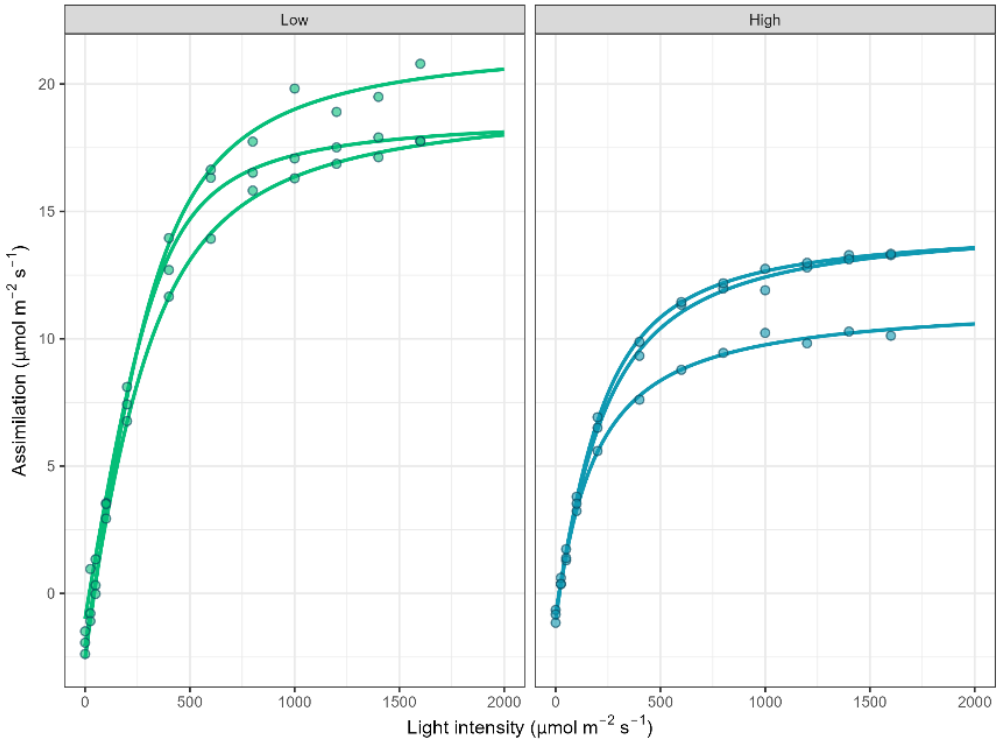

Supplementary Figure S3. Light response curves (*A/Q*) fitted by the *photosynthesis* package in R, for low density and high density grown *Wolffia australiana*. Points represent individual measurements and solid lines represent modelled AQ curves fitted to each replicate. n = 3.

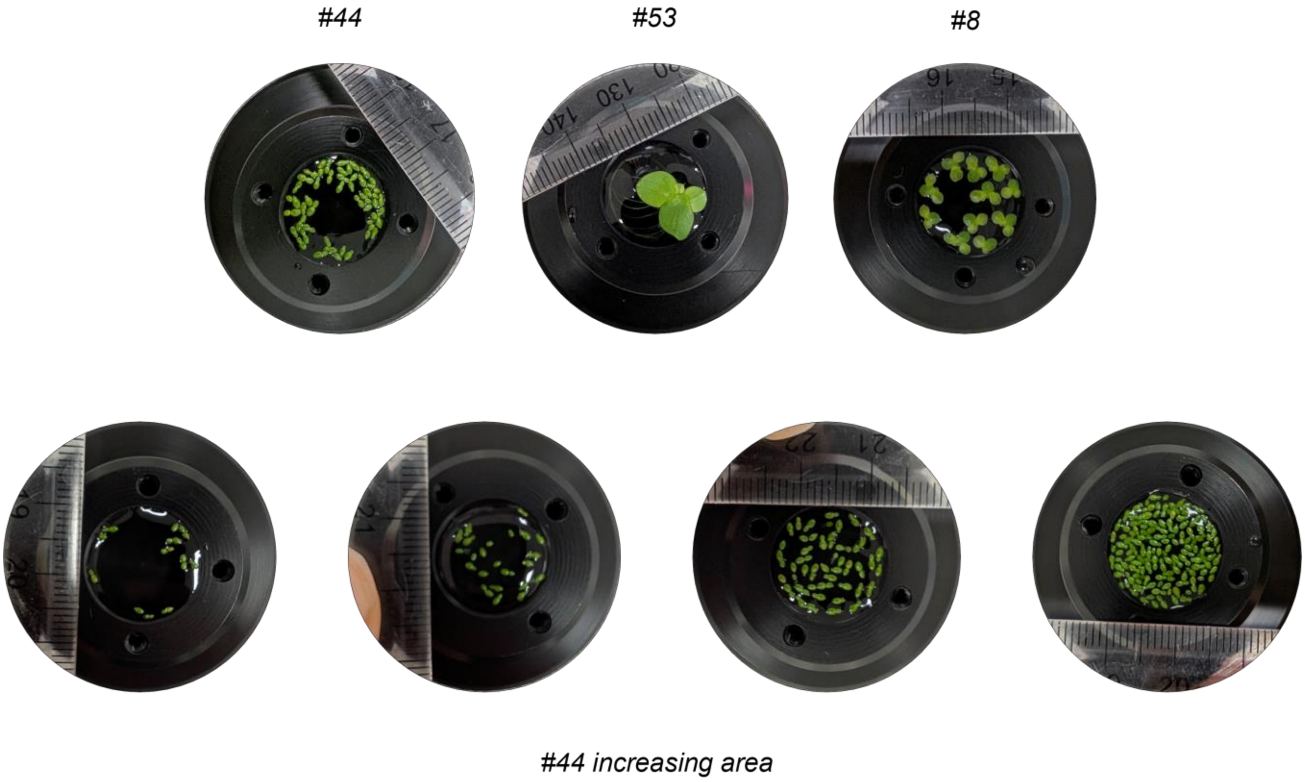

Supplementary Figure S4. Duckweed loaded into sample well adaptors. Top row shows representative samples for *Wolffia australiana* (#44), *Spirodela polyrhiza* (#53) and *Lemna minuta* (#8). Bottom row shows *Wolffia australiana* (#44) at increasing surface area/densities.

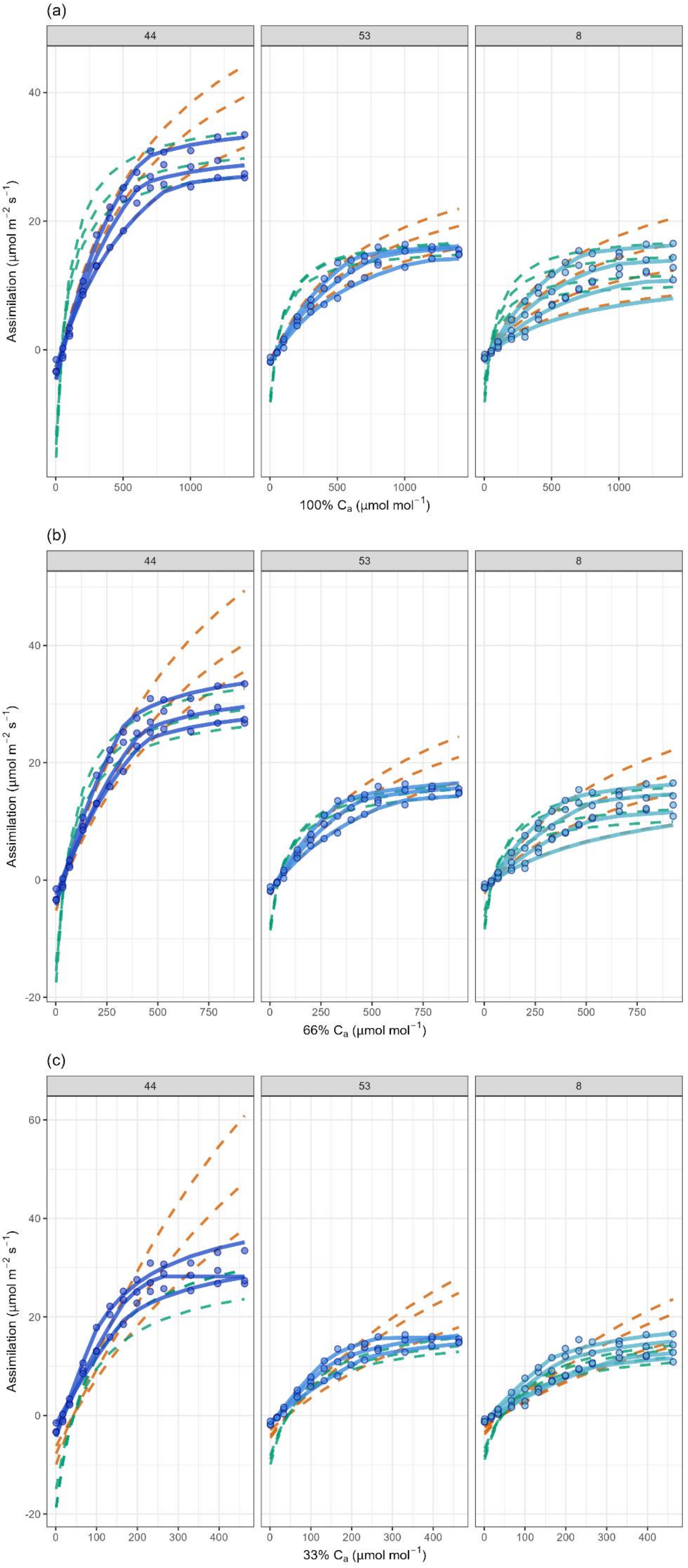
(a)
44
53
8
Assimilation (µmol m⁻² s⁻¹)
40
20
0
0
500
1000
100% Ca (µmol mol⁻¹)
(b)
44
53
8
Assimilation (µmol m⁻² s⁻¹)
40
20
0
-20
0
250
500
750
66% Ca (µmol mol⁻¹)
(c)
44
53
8
Assimilation (µmol m⁻² s⁻¹)
60
40
20
0
-20
0
100
200
300
400
33% Ca (µmol mol⁻¹)

Supplementary Figure S5. CO_2 response curves fitted by the *photosynthesis* package in R, for three duckweed species (44 = *Wolffia australiana*, 53 = *Spirodela polyrhiza*, 8 = *Lemna minuta*) at three estimated C_i values. C_i = (a) 100%, (b) 66% and (c) 33% of C_a. Points represent individual measurements and solid lines represent modelled curves fitted to each replicate. Dashed lines predict model CO_2 assimilation limited by the carboxylation capacity of Rubisco (green) and limited by the regeneration of RuBP (orange). n = 3.

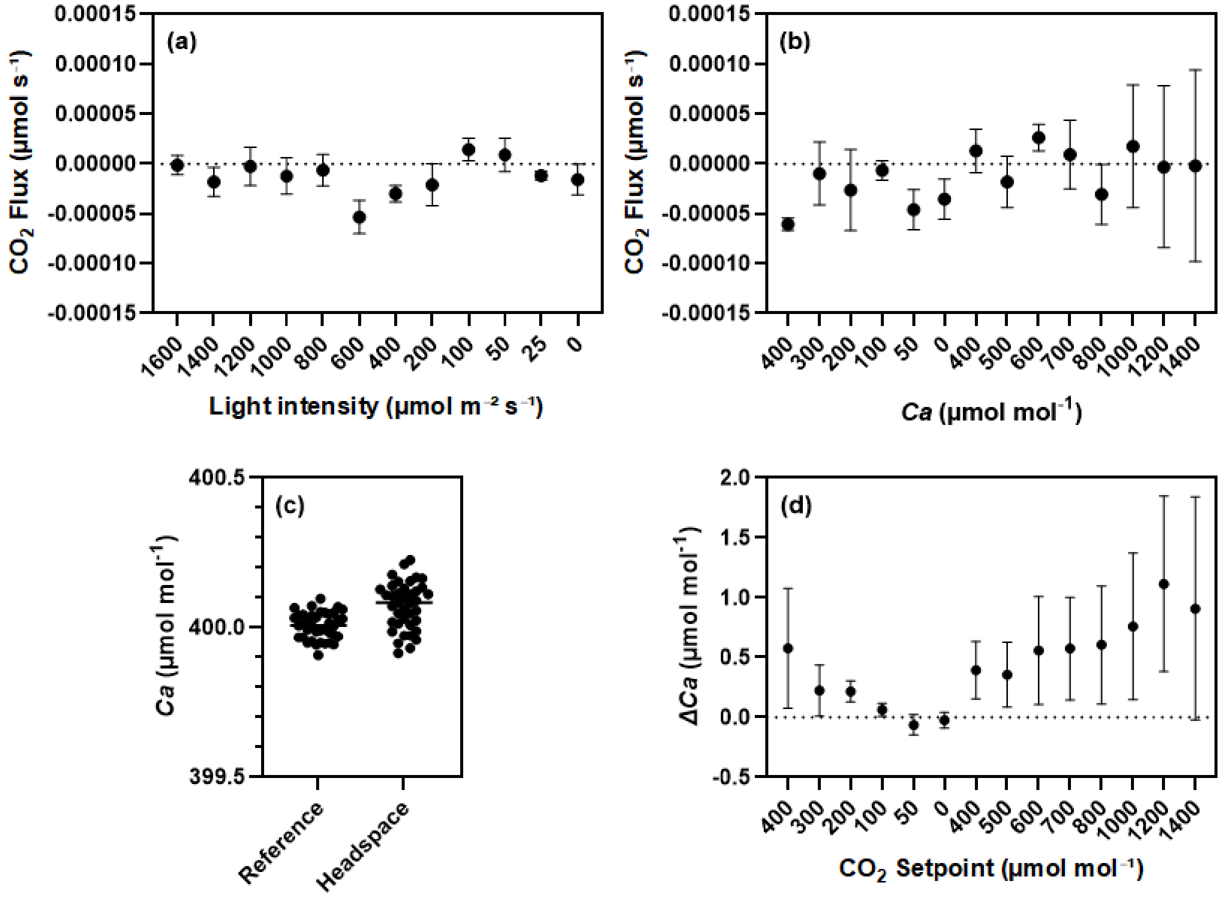

Supplementary Figure S6. N-Medium *only* optimisation runs. CO_2 assimilation during (a) light response curve (CO_2 reference: 400 µmol mol^{-1}) and (b) CO_2 response curve (Q_{sample} = 1000 µmol m^{-2} s^{-1}), n = 3, mean ± SEM. (c) CO_2 mole fraction reported by reference IRGA versus corrected headspace value during light response curve, n = 36, mean represented by straight line through data points. (d) Difference between corrected headspace CO_2 mole fraction and reported reference IRGA at each setpoint during CO_2 response curve, [ΔCa = corrected headspace Ca - CO_2 reference], n = 3, mean ± SEM.

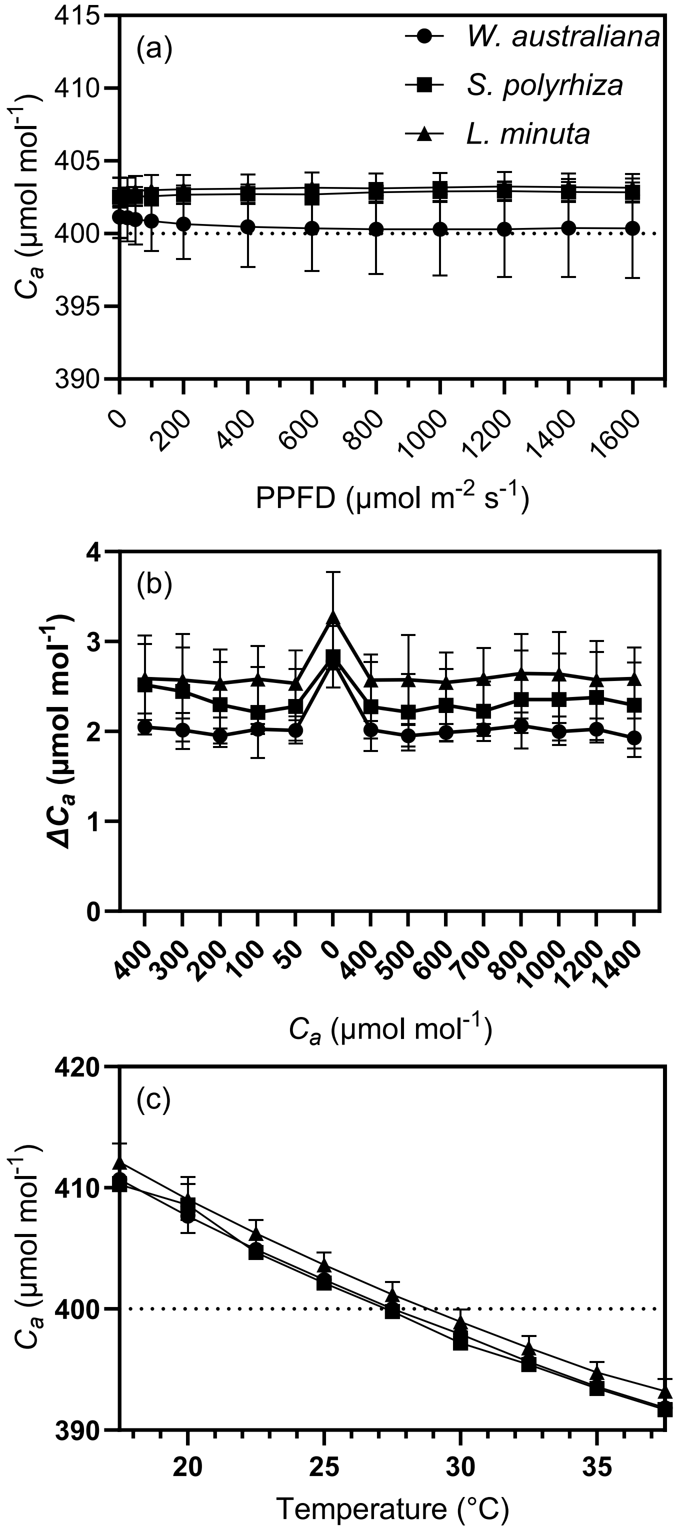

Supplentary Figure S7. Mean corrected headspace values for light, CO_2 and temperature response curves. (a) Light response (n = 5), (b) CO_2 response (n = 3-4), and (c) temperature response (n = 3) curves, shown as (a,c) absolute headspace CO_2 mole fraction or as (b) difference between corrected headspace CO_2 mole fraction and reported reference IRGA at each setpoint during CO_2 response curve. [ΔC_a = corrected headspace C_a - CO_2 reference]. Mean ± standard deviation.

Supplementary Table S1. Stomatal density in three duckweed species (*Wolffia australiana*, *Spirodela polyrhiza*, *Lemna minuta*). Data are mean ± SD, $n = 3$. One-way ANOVA (Tukey corrected for multiple comparisons was used to test species for each parameter), significance denoted by different lowercase letters.

Duckweed species	Stomatal density (stomata mm^{-2})
W. australiana	170 ± 19 a
S. polyrhiza	156 ± 21 a
L. minuta	207 ± 23 a

Supplementary Table S2. Q_{in} set points and respective Q_{sample} light intensities for light response curve, and combined gas exchange and fluorescence curve. As users can only control the light at Q_{in}, care must be taken to ensure that the Q_{sample} light environment is close to the users intended setpoints. Q_{in} setpoint and Red:Blue (R:B) ratio is rounded to two decimal places by the LI-COR software limiting users control over the final R:B ratio.

Light response curve - 90% Red light 10% Blue light at Q_{in}				
Q_{in} Setpoint (µmol m^{-2} s^{-1})	Q_{sample} Red (µmol m^{-2} s^{-1})	Q_{sample} Blue (µmol m^{-2} s^{-1})	Q_{sample} Total (µmol m^{-2} s^{-1})	Q_{sample} R : B ratio
0	0	0	0	0
46.55	22.6233	2.37405	24.99735	0.905 : 0.095
93.11	45.25146	4.74861	50.00007	0.905 : 0.095
186.22	90.50292	9.49722	100.00014	0.905 : 0.095
372.44	181.00584	18.99444	200.00028	0.905 : 0.095
744.88	362.01168	37.98888	400.00056	0.905 : 0.095
1117.32	543.01752	56.98332	600.00084	0.905 : 0.095
1489.76	724.02336	75.97776	800.00112	0.905 : 0.095
1862.2	905.0292	94.9722	1000.0014	0.905 : 0.095
2234.64	1086.03504	113.96664	1200.00168	0.905 : 0.095
2607.08	1267.04088	132.96108	1400.00196	0.905 : 0.095
2979.52	1448.04672	151.95552	1600.00224	0.905 : 0.095
Combined gas exchange and fluorescence – Blue light limited to 78.43 at Q_{in}				
Q_{in} Setpoint (µmol m^{-2} s^{-1})	Q_{sample} Red (µmol m^{-2} s^{-1})	Q_{sample} Blue (µmol m^{-2} s^{-1})	Q_{sample} Total (µmol m^{-2} s^{-1})	Q_{sample} R:B ratio
0	0	0	0	0
46.55	22.6233	2.37405	24.99735	0.905 : 0.095
93.11	45.25146	4.74861	50.00007	0.905 : 0.095
186.22	90.50292	9.49722	100.00014	0.905 : 0.095
372.44	181.00584	18.99444	200.00028	0.905 : 0.095
744.88	362.01168	37.98888	400.00056	0.905 : 0.095
1115.47	560.0016	39.9993	600.0009	0.933 : 0.067
1485.84	760.0014	39.9993	800.0007	0.95 : 0.05
1856.21	960.0012	39.9993	1000.0005	0.96 : 0.04
2226.58	1160.001	39.9993	1200.0003	0.967 : 0.033
2596.95	1360.0008	39.9993	1400.0001	0.971 : 0.029

2967.32	1560.0006	39.9993	1599.9999	0.975 : 0.025

Supplementary Dataset S1. Excel spreadsheet with all raw and normalised gas exchange data and duckweed areas.

Supplementary Dataset S2. *Photosynthesis* package arguments:

AQ curves were fitted using the fit_photosynthesis() function, with key arguments being

- .photo_fun = "aq_response"
- .model = "marshall_biscoe_1980"

From the *AQ* fits the following parameters were derived:

- Light compensation point, LCP = (Rd * (Rd * theta_J - k_sat)) / (phi_J * (Rd - k_sat))
- 90% of maximum assimilation, A_90 = (0.9 * (k_sat – Rd)) + Rd
- Light saturation point at 90% of maximum assimilation, LSP_90 = (A_90 * (k_sat - theta_J * A_90)) / (phi_J * (k_sat - A_90))

ACi curves were fitted using fit_Aci_response(), with the default arguments. *AT* curves were fir using the fit_t_response() function, with key arguments being

- model = "Topt"
- start = list(Ea = 60000, Par25 = [assimilation at 25°C], Hd = 150000)
